# An Update on Adipose‐Derived Stem Cells for Regenerative Medicine: Where Challenge Meets Opportunity

**DOI:** 10.1002/advs.202207334

**Published:** 2023-05-10

**Authors:** Yi Qin, Gaoran Ge, Peng Yang, Liangliang Wang, Yusen Qiao, Guoqing Pan, Huilin Yang, Jiaxiang Bai, Wenguo Cui, Dechun Geng

**Affiliations:** ^1^ Department of Orthopaedics The First Affiliated Hospital of Soochow University Orthopaedic Institute, Medical College Soochow University Suzhou Jiangsu 215006 China; ^2^ Department of Orthopaedics The Affiliated Changzhou No. 2 People's Hospital of Nanjing Medical University Changzhou Jiangsu 213000 China; ^3^ Institute for Advanced Materials School of Materials Science and Engineering Jiangsu University Zhenjiang Jiangsu 212013 China; ^4^ Department of Orthopaedics Shanghai Key Laboratory for Prevention and Treatment of Bone and Joint Diseases Shanghai Institute of Traumatology and Orthopaedics Ruijin Hospital Shanghai Jiao Tong University School of Medicine Shanghai 200025 China

**Keywords:** 3D bioprinting, adipose‐derived stem cells, cell‐free therapy, clinical application, regenerative medicine

## Abstract

Over the last decade, adipose‐derived stem cells (ADSCs) have attracted increasing attention in the field of regenerative medicine. ADSCs appear to be the most advantageous cell type for regenerative therapies owing to their easy accessibility, multipotency, and active paracrine activity. This review highlights current challenges in translating ADSC‐based therapies into clinical settings and discusses novel strategies to overcome the limitations of ADSCs. To further establish ADSC‐based therapies as an emerging platform for regenerative medicine, this review also provides an update on the advancements in this field, including fat grafting, wound healing, bone regeneration, skeletal muscle repair, tendon reconstruction, cartilage regeneration, cardiac repair, and nerve regeneration. ADSC‐based therapies are expected to be more tissue‐specific and increasingly important in regenerative medicine.

## Graphical abstract

Adipose‐derived stem cells (ADSCs) serve as an ideal candidate for regenerative medicine owing to their ability to differentiate into multilineages, facilitate angiogenesis, suppress apoptosis, and participate in immunoregulation. Numerous preclinical studies and clinical trials have demonstrated the therapeutic potential of ADSCs in fat grafting, wound healing, bone regeneration, skeletal muscle repair, tendon reconstruction, cartilage regeneration, cardiac repair, and nerve regeneration.

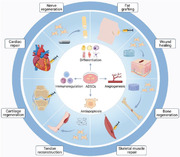

## 1 Introduction

The past few decades have witnessed exponential growth in regenerative medicine. As an important branch of regenerative medicine, stem cell‐based therapy opens a new avenue for patients with incurable diseases for whom conventional treatments fail. The goal of stem cell‐based therapy is to rejuvenate or replace dysfunctional tissues and organs through stem cell pluripotency, self‐renewal, and regenerative cytokine secretion.^[^ ^1^ ^]^ In general, stem cells are divided into three categories: embryonic stem cells, induced pluripotent stem cells, and adult stem cells. The first challenge in stem cell‐based therapy is to identify appropriate sources of cells as regenerative agents. Embryonic stem cells are naturally pluripotent; however, their allogeneic sources and moral concerns restrict their application. Induced pluripotent stem cells were initially generated by reprogramming with four specific genes (*OCT4*, *SOX2*, *KLF4*, and *C‐MYC*) and have been applied in disease modeling and drug discovery.^[^ ^2^ ^]^ However, induced pluripotent stem cells tend to differentiate toward immature embryonic or fetal state rather than fully mature adult cells. In addition, the low induction rate and unclear underlying molecular mechanisms remain obstacles to their clinical application.^[^ ^3^ ^]^

As the most frequently described population of adult stem cells, mesenchymal stem cells (MSCs) serve as an ideal candidate for regenerative medicine owing to their ability to self‐renew and differentiate into tissue‐specific cells. MSCs are present in the umbilical cord blood,^[^ ^4^ ^]^ placenta,^[^ ^5^ ^]^ muscle,^[^ ^6^ ^]^ and other tissues, with bone marrow and adipose tissue being the most important sources. The International Society for Cellular Therapy (ISCT) published three minimum criteria for defining MSCs in 2006: adhesion to a plastic surface under standard culture conditions; the ability to differentiate into osteoblasts, adipocytes, and chondroblasts in vitro; and phenotypically, CD105, CD73, and CD90 positivity and negativity for CD45, CD34, CD14 or CD11b, CD79a or CD19, and HLA‐DR.^[^ ^7^ ^]^ Bone marrow‐derived MSCs (BM‐MSCs) are the most widely studied MSCs. However, the harvesting of bone marrow is a highly invasive procedure, and the number of the MSCs obtained from bone marrow is limited,^[^ ^8^ ^]^ so an alternative stem cell source for BM‐MSCs should be identified.

In 2001, adipose‐derived stem cells (ADSCs) were first isolated from the stromal vascular fraction (SVF) obtained by processing adipose tissue.^[^ ^9^ ^]^ As the largest endocrine tissue that regulates metabolism and the immune system, adipose tissue is omnipresent in humans.^[^ ^10^ ^]^ Adipose tissues are generally classified as white or brown adipose tissue, which differ considerably in morphology, function, and metabolic activity. White adipose tissue is predominantly involved in storing excess energy as lipids and serves as a source of ADSCs in most studies.^[^ ^11^ ^]^ Brown adipose tissue (BAT) has been thought to exist only in infants and decline with increasing age. However, recent studies have demonstrated the presence of functional BAT in adult humans, distributed in cervical, supraclavicular, mediastinal, paravertebral, and suprarenal regions.^[^ ^12^ ^]^ The existence of ADSCs within BAT has also been demonstrated. Compared with white adipose tissue‐derived ADSCs, BAT‐derived ADSCs exhibited a similar multilineage differentiation potential, with an enhanced capacity to differentiate into active brown adipocytes.^[^ ^12a^ ^]^ Given the key role of BAT in systemic energy homeostasis, BAT‐derived ADSCs may have therapeutic potential for the treatment of obesity and metabolic disorders. ADSCs are not a homogenous cell population, and many researchers have attempted but failed to identify a specific CD marker to characterize ADSCs explicitly. ISCT identified surface markers of ADSCs in 2013, including CD13^+^/CD29^+^/CD44^+^/CD73^+^/CD90^+^/CD105^+^ and CD31^‐^/CD45^‐^/CD235a^‐^.^[^ ^13^ ^]^ It is worth noting that the phenotype of ADSCs during culture is dynamic; some markers are expressed de novo, while the expression of others is lost.^[^ ^14^ ^]^ For example, CD34 has been demonstrated to be at its peak levels in early‐passage ADSCs, whereas its expression decreases throughout the culture period.^[^ ^15^ ^]^

**Figure 1:**
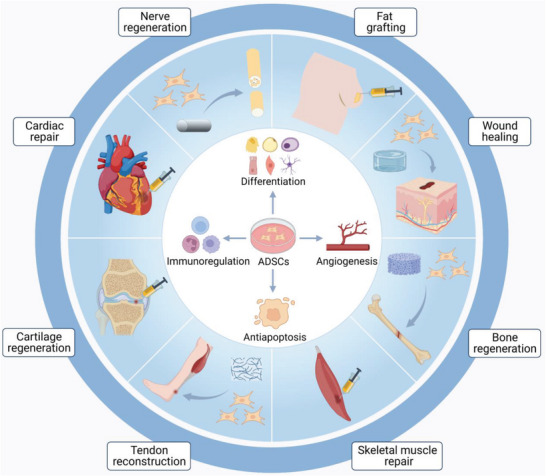
Schematic representation of adipose‐derived stem cells (ADSCs) for regenerative therapies. ADSCs serve as an ideal candidate for regenerative medicine owing to their ability to differentiate into multilineages, facilitate angiogenesis, suppress apoptosis, and participate in immunoregulation. Numerous preclinical studies and clinical trials have demonstrated the therapeutic potential of ADSCs in fat grafting, wound healing, bone regeneration, skeletal muscle repair, tendon reconstruction, cartilage regeneration, cardiac repair, and nerve regeneration.

ADSCs demonstrate morphological and immunophenotypic characteristics of MSCs.^[^ ^16^ ^]^ The great advantage of ADSCs is that they can be harvested through a less invasive method and in larger quantities without any ethical concerns. Since their discovery, ADSCs have become a research hotspot with promising prospects in regenerative medicine. In this review, we highlight current challenges in translating ADSC‐based therapies into clinical settings, discuss novel strategies for overcoming the limitations of ADSCs, and provide an update on the advancements in ADSCs for regenerative medicine (**Figure** **1**). Therefore, the purpose of this review is not only to emphasize the challenges and opportunities in this emerging field, but also to further establish ADSC‐based therapies as an emerging platform for regenerative medicine.

## 2 Therapeutic Potential of ADSCs and Current Challenges

Regenerative medicine aims at repairing or replacing tissues damaged by genetic, traumatic, or degenerative defects. This rationale is based either on the ability of stem cells to replace dead cells with newly differentiated progenies, or on their paracrine activity, which actively promotes tissue regeneration. When administrated directly or in combination with biofunctional scaffolds, ADSCs repopulate damaged tissues via adhesion, proliferation, and differentiation.^[^ ^17^ ^]^ ADSCs are MSCs of mesodermal origin with the capacity for classical adipogenic, osteogenic, and chondrogenic differentiation. In a donor‐matched comparison between ADSCs and BM‐MSCs, ADSCs exhibited higher proliferation and adipogenic capacities, while BM‐MSCs showed higher osteogenic and chondrogenic capacities, indicating that MSCs may be more steered toward certain lineage differentiation depending on their tissue origin.^[^ ^18^ ^]^ However, studies have also suggested that the osteogenic differentiation capability of ADSCs is comparable to that of BM‐MSCs.^[^ ^19^ ^]^ These opposite results may attribute to donor variation and heterogeneity among different MSC populations. Interestingly, ADSCs can also differentiate into nonmesenchymal cell lineages, such as endothelial, myogenic, and neuronal lineages.^[^ ^20^ ^]^ The differentiation of ADSCs toward specific cell types is regulated by a combination of several inductive factors. Hence, cell culture media containing lineage‐specific induction factors, mechanical or electromagnetic stimulation, and genetic reprogramming have been widely used to induce the differentiation of ADSCs into specific cell types. This multipotent property makes ADSCs a promising candidate for tissue regeneration.

However, the therapeutic potential of ADSCs is not limited to cell replacement. ADSCs have paracrine activity and secrete a broad spectrum of bioactive molecules, such as cytokines, antioxidant factors, chemokines, and growth factors.^[^ ^21^ ^]^ The paracrine mechanism plays crucial roles in various therapeutic effects, including facilitating angiogenesis, suppressing apoptosis, and participating in immunoregulation. Vascularization of newly formed tissues can supply sufficient nutrients and oxygen, and transport waste and possible material degradation products. Hence, angiogenic processes are necessary for the survival of the regenerated tissue. Vascular endothelial growth factor (VEGF), transforming growth factor‐*β* (TGF‐*β*), platelet‐derived growth factor (PDGF), angiogenin (ANG), and other angiogenic cytokines secreted by ADSCs can synergistically promote the angiogenic process and facilitate tissue regeneration.^[^ ^22^ ^]^ In addition, ADSCs exert an anti‐apoptotic effect via the secretion of insulin‐like growth factor‐1 (IGF‐1) and exosomes, protecting both damaged native tissues and newly regenerated tissues.^[^ ^23^ ^]^ Immunoregulation is another mechanism through which ADSCs exhibit therapeutic potential. Inflammation is generally present in damaged tissues, without which the initiation and completion of the repair process cannot occur. However, excessive inflammation impairs tissue regeneration. ADSCs can suppress dendritic cell differentiation, immunoglobulin synthesis, CD8^+^ and CD4^+^ T lymphocytes and natural killer cell proliferation, and promote M2 macrophage polarization and regulatory T‐cell proliferation.^[^ ^24^ ^]^ ADSCs can alleviate excessive inflammation and regulate the immune system through direct cell–cell contact or indirect paracrine activity, thereby facilitating tissue regeneration.^[^ ^25^ ^]^

Despite the recent encouraging outcomes of preclinical and clinical studies on ADSCs in recent years, concerns have arisen that ADSCs transplantation may increase the risk of tumor growth and metastasis.^[^ ^26^ ^]^ The therapeutic potential of ADSCs is primarily attributed to their roles in proangiogenesis, cellular homing, and immune regulation. These properties may induce tumor progression through similar mechanisms that promote tissue regeneration. ADSCs can be recruited to tumors and integrated into the tumor stroma, after which some ADSCs are converted to cancer‐associated fibroblasts, while others remain as ADSCs.^[^ ^27^ ^]^ Cancer‐associated fibroblasts are integral components of the tumor microenvironment, which can promote cancer cell proliferation, immune exclusion, and therapy resistance by secreting growth factors, inflammatory ligands, and extracellular matrix proteins.^[^ ^28^ ^]^ Meanwhile, undifferentiated ADSCs in tumors exhibit active paracrine activity and secrete various cytokines that facilitate tissue regeneration, including growth factors and VEGF. These bioactive molecules secreted into the tumor microenvironment may enhance tumor vascularization, promote the survival and proliferation of tumor cells, and accelerate tumor progression.^[^ ^29^ ^]^ The potential tumorigenicity of ADSCs has been widely discussed in the field of breast reconstruction because the breast microenvironment after oncological mastectomy is completely different from that of the normal microenvironment.^[^ ^30^ ^]^ Koellensperger et al. evaluated the interactions between ADSCs and five human breast cancer cell lines (BRCAs), including MCF‐7, MDA‐MB‐231, SK‐BR‐3, ZR‐75‐30, and EVSA‐T.^[^ ^31^ ^]^ Their results revealed that ADSCs could enhance multiple malignant features of BRCAs in vitro, such as gene expression, protein secretion, migration, and angiogenesis. In addition, Goto et al. proposed that ADSCs isolated from patients with breast cancer can promote human breast cancer patient‐derived xenograft tumor growth through the adipokine adipsin, which serves as a component of the tumor microenvironment in breast cancers.^[^ ^32^ ^]^

In contrast, a recent study by Ejaz et al. showed that ADSCs do not increase the proliferation rate of breast cancer cells either through paracrine secretion or contact‐dependent interactions.^[^ ^33^ ^]^ The authors also emphasized the importance of studies in appropriate animal models and long‐term clinical data from postoperative patients, as the majority of experimental studies utilized ADSCs and in vitro differentiated adipocytes as models,^[^ ^31^, ^32^, ^34^ ^]^ which may not reflect the actual clinical scenario. Although discussions on the potential tumorigenicity of ADSCs are ongoing, there is no doubt that large randomized and controlled clinical trials are essential to reach a final safety recommendation.

Another challenge in the clinical translation of ADSC‐based therapies is the uncertainty regarding their clinical efficacy. Most of our understanding of ADSCs today comes from studies performed on in vitro 2D cell culture systems, which generally ignore critical characteristics such as cell–cell and cell–extracellular cell matrix (ECM) interactions, tissue architecture, and biophysical cues of the 3D niche. Although animal models have been employed in preclinical studies to evaluate the efficacy and safety of therapies, researchers have found it difficult to apply their success in clinical settings because of the lack of physiological, molecular, and genetic relevance to human clinical conditions.^[^ ^35^ ^]^ Worse still, the absence of standard procedures for applying ADSCs leads to varied cell qualities, which increases the uncertainty of their clinical efficacy. The intrinsic characteristics of donors can influence the properties of isolated ADSCs. For example, increasing the age of donors may reduce the proliferation and differentiation potential of ADSCs,^[^ ^36^ ^]^ whereas studies suggest no significant impact of sex.^[^ ^37^ ^]^ Moreover, ADSCs obtained from obese donors exhibited increased proliferation and migration capacity, excessive immune response, and decreased differentiation potential. These properties are modulated by the microenvironment within the adipose tissue, which is characterized by low oxygen levels and chronic inflammation in the obese context.^[^ ^38^ ^]^ Similarly, the detrimental effects of diabetes and radiotherapy on ADSCs have been demonstrated.^[^ ^39^ ^]^ In addition, there is depot‐dependent variability in the properties of ADSCs population. Compared to ADSCs harvested from the omentum, those from subcutaneous and intrathoracic adipose tissues exhibited higher adipogenic differentiation potential but lower osteogenic differentiation capacity.^[^ ^40^ ^]^ Different sources and a lack of standard isolation procedure result in varied ADSC qualities, making it difficult to compare different experimental results. Although ADSCs appear to be the most advantageous cell type for regenerative therapies, the clinical translation of ADSC‐based therapies remains unclear.

## 3 Novel Strategies for Overcoming the Limitations of ADSCs

### 3.1 ADSCs Secretome and Cell‐Free Therapy

As mentioned above, ADSCs exert their therapeutic effect not only by direct cell‐to‐cell interactions, but also through the secretome, including extracellular vesicles (EVs), cytokines, and other active substances. Hence, using the ADSCs secretome to enhance tissue regeneration may be a promising alternative to conventional ADSCs therapies.^[^ ^41^ ^]^ The cell‐free therapy utilizing the ADSCs secretome avoids many shortcomings of administering whole cells, such as potential tumorigenicity and storage problems. Compared with ADSCs, the secretome is more stable and less immunogenic, making it a better candidate for allogeneic transplantation and commercial promotion.^[^ ^42^ ^]^

In the cell culture process under laboratory conditions, ADSCs tend to release a broad spectrum of bioactive factors into the culture medium, which is termed conditioned medium (CM). ADSC‐CM comprises the entire ADSCs secretome and has been demonstrated to exert beneficial effects on wound healing,^[^ ^43^ ^]^ cardioprotection,^[^ ^44^ ^]^ and neuroprotection.^[^ ^45^ ^]^ EVs are a heterogeneous group of cell‐derived membranous structures that play a crucial role in the therapeutic potential of ADSC‐CM and consist of various subclasses including exosomes, microvesicles, and apoptotic bodies.^[^ ^46^ ^]^ In recent years, ADSC‐derived exosomes (ADSC‐Exos) harvested from ADSC‐CM have attracted research interest in regenerative therapies.

**Figure 2:**
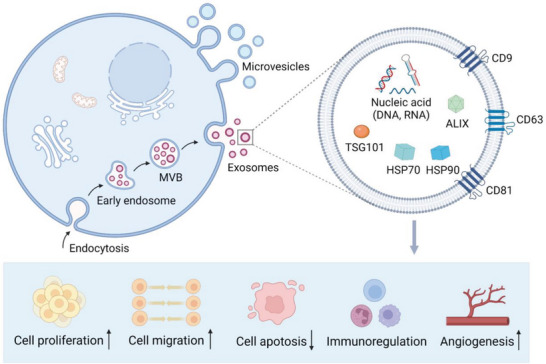
Schematic illustration of the biogenesis and therapeutic potential of adipose‐derived stem cell‐derived exosomes (ADSC‐Exos). The process begins with endocytosis and the formation of early endosomes, which can mature into multivesicular bodies (MVBs) with the accumulation of intraluminal vesicles (ILVs). When MVBs fuse with the plasma membrane, ILVs are released as exosomes. ADSC‐Exos contain tetraspanins (CD9, CD63, and CD81), biogenesis‐related proteins (TSG101 and ALIX), heat shock proteins (HSP70 and HSP90) and nucleic acids (DNA, mRNA, miRNA, and rRNA). ADSC‐Exos exhibit therapeutic potential by enhancing cell proliferation, cell migration, and angiogenesis, participating in immunoregulation, and suppressing apoptosis.

Exosomes are small EVs with diameters of 40–160 nm.^[^ ^47^ ^]^ The biogenesis begins with endocytosis and the formation of early endosomes, which mature into multivesicular bodies (MVBs) with the accumulation of intraluminal vesicles (ILVs). ILVs are released as exosomes through the fusion of MVBs with the plasma membrane.^[^ ^47^, ^48^ ^]^ Several proteins are enriched in the ADSC‐Exos, such as tetraspanins (CD9, CD63, and CD81), biogenesis‐related proteins (TSG101 and ALIX), and heat shock proteins (HSP70 and HSP90).^[^ ^49^ ^]^ In addition, ADSC‐Exos contain nucleic acids including DNA, mRNA, miRNA and rRNA.^[^ ^50^ ^]^ Using these varied proteins and genetic materials, ADSC‐Exos are involved in cell proliferation, migration, apoptosis, regulation of immune and inflammatory responses, and promotion of angiogenesis (**Figure** **2**).^[^ ^51^ ^]^ In comparison to their parent cells, certain components are enriched in ADSC‐Exos, while others are present in reduced quantities.^[^ ^46^ ^]^ Despite the differences in composition, Chen et al. noted that ADSC‐Exos were comparable to source ADSCs in terms of achieving improved fat graft retention, suggesting that ADSC‐Exos are equally potent when administered in vivo.^[^ ^52^ ^]^ To date, the regenerative effects of ADSC‐Exos have been confirmed in numerous fields, including wound healing,^[^ ^53^ ^]^ bone regeneration,^[^ ^54^ ^]^ cartilage repair,^[^ ^55^ ^]^ myocardial protection,^[^ ^56^ ^]^ and nerve regeneration.^[^ ^57^ ^]^

The more effective application of ADSC‐Exos in regenerative therapy has attracted considerable attention. Engineering ADSC‐Exos with a specific therapeutic cargo to incorporate the desired functionalities may be a feasible strategy for ADSC‐Exo applications. Chen et al. harvested exosomes from ADSCs overexpressing miR‐375 after lentiviral transfection. When implanted into a rat calvarial defect model, ADSC‐Exos enriched with miR‐375 intensified bone formation more effectively than those unmodified.^[^ ^58^ ^]^ In addition, Han et al. revealed that exosomes from hypoxia‐treated ADSCs exhibited a higher capacity to enhance angiogenesis than those from normoxia‐treated ADSCs, demonstrating that the biological activity of ADSC‐Exos can be altered by the culturing conditions.^[^ ^59^ ^]^ Furthermore, incorporating ADSC‐Exos into a suitable biomaterial has the potential to control their release, distribution, and retention in vivo, thus serving as a promising alternative method of administration.^[^ ^60^ ^]^

Unlike MVB‐derived exosomes, microvesicles are generated by outward budding and fission of the plasma membrane, which is the result of dynamic interplay between phospholipid redistribution and cytoskeletal protein contraction. The formation of microvesicles is initiated by the activity of aminophospholipid translocases that transfer phospholipids from one leaflet of the plasma membrane to the other. ADP‐ribosylation factor 6 plays an important role in microvesicles budding by stimulating phospholipase D activity, which promotes the activation of extracellular signal‐regulated kinase (ERK). The contractile protein myosin light streptokinase is then phosphorylated by ERK, leading to the serine phosphorylation of myosin II, ultimately triggering the release of microvesicles.^[^ ^61^ ^]^ ADSC‐derived microvesicles (ADSC‐MVs) tend to be larger in size (100–1000 nm) relative to ADSC‐Exos, though the size ranges overlap between these two types of EVs. Like ADSC‐Exos, ADSC‐MVs transport multiple proteins and nucleic acids to act as enhancers for tissue regeneration.^[^ ^62^ ^]^

Apoptosis is a form of programmed cell death that begins with condensation of the nuclear chromatin, followed by membrane blebbing, and progresses to disintegration of the cellular contents. Apoptotic bodies are membrane enclosed vesicles generated during the final stages of apoptosis and are extremely heterogeneous in size, ranging from 500 to 4000 nm in diameter.^[^ ^63^ ^]^ A wide variety of functional biomolecules are present in apoptotic bodies, making them a promising candidate for regenerative medicine.^[^ ^64^ ^]^ To date, studies focusing on ADSC‐derived apoptotic bodies (ADSC‐Abs) are limited, whereas a recent study explored the promoting effect of ADSC‐Abs on wound healing.^[^ ^65^ ^]^ Further studies are required to evaluate their efficacy in tissue regeneration.

ADSCs secretome has been proposed as a regenerative mediator for cell‐free therapy with increased effectiveness and fewer side effects. Nevertheless, several challenges remain in this emerging field. Differential ultracentrifugation is the most widely used approach for EV isolation because of its relatively simple protocol and high yield, but this method cannot distinguish EVs with overlapping ranges, such as exosomes and microvesicles.^[^ ^66^ ^]^ Multiple other isolation methods have been developed, such as density gradient ultracentrifugation, size‐exclusion chromatography, and affinity capture.^[^ ^67^ ^]^ However, it is still difficult to balance the purity, yield, and cost of EVs. Due to the various qualities of EVs arising from the lack of standard isolation methods, data from different studies seem incomparable. Furthermore, demonstrating a function specific to a given EV source is challenging for researchers in this field. The following demonstrations are required according to the minimal information for studies of extracellular vesicles 2018 (MISEV2018) position paper: 1) the activity is observed in the absence of direct cell–cell contact; 2) the activity is predominantly associated with EVs rather than with soluble mediators; 3) the activity is closely related to EVs rather than with co‐isolated components; 4) the function is specific to a given EV source, as compared with other EVs with similar diameters; and 5) the function is mediated by a specific component in EVs rather than others.^[^ ^68^ ^]^ Only in this way can the specific mechanism underlying the regenerative effect of EVs be fully elucidated. In addition, multiple injections are generally required to maintain the desired effects in cell‐free therapy because the drugs used do not contain stem cells, which may reduce patient compliance and increase the cost of treatment.^[^ ^69^ ^]^ Further systematic and rigorous research to determine the therapeutic potential of EVs will drive evidence‐based applications of cell‐free therapy.

### 3.2 3D Bioprinting Using ADSCs in Regenerative Medicine

**Figure 3:**
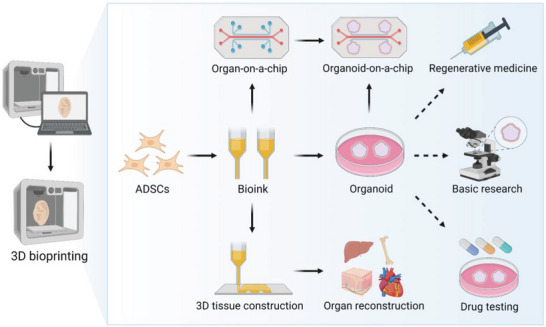
Application of 3D bioprinting for organoid production and organ reconstruction. Bioinks containing ADSCs are printed as organoids, organ‐on‐a‐chip systems, and organ‐level 3D tissues. Organoids have been widely used in basic research, drug testing, and regenerative medicine. The Organoids‐on‐a‐chip technology combines the best features of organoids and organ‐on‐a‐chip systems, becoming a promising approach for recapitulating the key aspects of human physiology and pathophysiology.

3D bioprinting is a state‐of‐the‐art strategy for recapitulating the functional organization of human tissues and 3D micro‐tissue environments. The bioinks used in 3D bioprinting technology are composed of living cells, base structural materials, and other essential components.^[^ ^70^ ^]^ Depending on the computer‐aided design and automated dispensing systems, bioprinting can precisely control the deposition of cells and biomatrices in a layer‐by‐layer manner to fabricate biomimetic tissue constructs or even functional organs.^[^ ^71^ ^]^ Therefore, 3D bioprinting has become a major research topic in recent years and facilitated the development of numerous technologies, including organoids, organ‐on‐a‐chip systems, and 3D tissue construction, providing new opportunities for the widespread use of ADSCs in regenerative medicine (**Figure** **3**).

Recent advances in 3D cell culture technologies have played a crucial role in organoid culture. Organoids are complex 3D structures grown from stem cells through a self‐organization process that display functionalities and architectures similar to in vivo organs.^[^ ^72^ ^]^ Numerous studies have demonstrated the potential use of organoids in disease modeling, drug screening, and tissue engineering. However, their variability and limited scale of organoid restrict their application.^[^ ^73^ ^]^ Recently, Lawlor et al. applied automated extrusion‐based 3D bioprinting to manufacture kidney organoids.^[^ ^74^ ^]^ Compared with the manual organoid generation, organoid bioprinting increased the throughput of manufacturing nine‐fold with highly reproducible cell number, diameter, and cell viability. Additionally, Brassard et al. generated centimeter‐scale tissues through organoid bioprinting, which comprised branched vasculature, lumens, and tubular intestinal epithelia with villus domains and crypts.^[^ ^75^ ^]^ This study revealed that organoid technology and bioprinting can be merged to achieve controllable complex biomimetic structures. The combination of 3D bioprinting and organoid production overcomes the limitations of traditional organoid generation and facilitates their application in basic research, drug testing, and regenerative medicine. 3D bioprinting using organoid‐forming ADSCs may expand the prospects of ADSC‐based therapies in the near future.

The organ‐on‐a‐chip system is another microphysiological system consisting of transparent 3D polymeric microchannels lined with living human cells.^[^ ^76^ ^]^ Compared with conventional soft lithography, 3D bioprinting can be used to easily design and construct complex microfluidic systems, providing a more efficient choice for fabricating biological structures with heterogeneity, 3D cell distribution, and tissue‐specific functions.^[^ ^77^ ^]^ Recently, an emerging technology called organoids‐on‐a‐chip has become a promising approach for recapitulating key aspects of human physiology and pathophysiology. Organoids‐on‐a‐chip technology combines the best features of organoids with those of organ‐on‐a‐chip systems. This platform can control organoids in the microenvironment and model interactions between tissues and organs.^[^ ^78^ ^]^ Although this technology is still in its infancy, we expect that organoids‐on‐a‐chip with 3D bioprinting will become an important complement to current animal models and facilitate the clinical translation of ADSC‐based therapies.

3D bioprinting using ADSCs has achieved initial success in 3D tissue construction and organ reconstruction. Sorkio et al. produced 3D cornea‐mimicking tissues by using ADSCs and laser‐assisted 3D bioprinting.^[^ ^79^ ^]^ In this study, ADSCs were used as a cell source for constructing stroma‐mimicking structures and organized horizontally as in the native corneal stroma. When implanted into organ‐cultured porcine corneas, the 3D bioprinted stromal structures exhibited attachment to the host tissue with signs of ADSCs migration from the printed structure. Lee et al. used bioprinting technology including a sacrificial layer process to fabricate a dual‐cell‐type printed structure with an ear shape.^[^ ^80^ ^]^ In the main part, adipocytes and chondrocytes differentiated from ADSCs were encapsulated in hydrogel to dispense into the fat and cartilage regions of ear‐shaped structures. The artificial ears satisfied the expectations for both the appearance and anatomy of the native ear, indicating the feasibility of bioprinting for regenerating organs with complex shapes. Taken together, the introduction of 3D bioprinting in the field of ADSC‐based therapies allows us to mimic the complexity of human tissue and represents a significant step toward organ‐level functional 3D tissue construction.

### 3.3 Optimized and Standardized Procedures for Applying ADSCs

**Figure 4:**
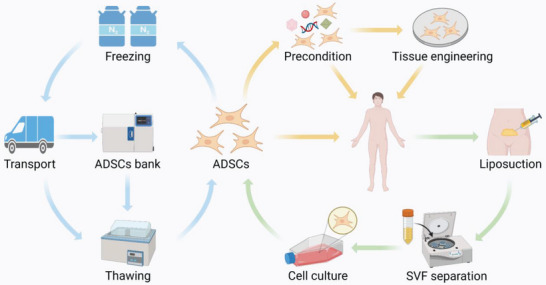
Procedures for applying adipose‐derived stem cells (ADSCs). Green arrows indicate the isolation of ADSCs. Adipose tissues are harvested from healthy donors or patients using liposuction and processed using enzymatical or mechanical procedures to separate stromal vascular fraction (SVF). Then ADSCs are obtained through seeding SVF in culture. Yellow arrows indicate the clinical use of ADSCs. According to the damaged organs of patients, ADSCs can be directly administered, preconditioned to enhance therapeutic effects, or combined with biomaterials for tissue engineering. Blue arrows indicate the storage of ADSCs. Cryopreserved ADSCs can be transported to medical institutions at a distance or ADSCs banks for long‐term storage until needed.

Although ADSCs appear to meet the criteria for ideal cell therapy in regenerative medicine, the absence of standard protocols for ADSCs application limits their widespread clinical use. ADSCs isolated from different donors and depots exhibit different proliferative, migratory, and differentiation properties. Most of the present clinical trials on ADSCs have applied autologous or allogeneic ADSCs obtained from subcutaneous adipose tissue because they can be easily harvested through liposuction. A deeper understanding of the properties of ADSCs from different donors and depots is important for clinical applications, which would provide crucial information on suitable patient selection, optimal timing of ADSCs harvesting, and expectations for clinical curative effects. In addition, different isolation methods can affect the yield and biology of ADSCs. Optimized and standardized procedures for applying ADSCs at different checkpoints are essential for translating ADSC‐based therapies to the clinic (**Figure** **4**).

Although the harvesting procedure described by Zuk et al. in 2001 remains the most commonly used method for SVF processing and ADSCs isolation,^[^ ^9^ ^]^ many modified methods have been proposed. In terms of the yield and biology of ADSCs, power‐assisted liposuction is preferable to other harvesting techniques, such as surgical resection and laser‐assisted liposuction.^[^ ^81^ ^]^ Shah et al. reported a new method for isolating ADSCs from lipoaspirates without enzymatic treatment. Instead of expensive collagenase, this simple washing method yields a similar cell product with a favorable immunophenotype.^[^ ^82^ ^]^ Palumbo et al. compared the spontaneous lipoaspirate stratification (10, 20, or 30 min) to the centrifugation technique (90, 400, or 1500×*g*) and analyzed the yield of ADSCs.^[^ ^83^ ^]^ Their results indicated that spontaneous stratification at 20 min or centrifugation at 400×g can provide sufficient ADSCs and preserve adipocyte integrity, demonstrating the effectiveness of both approaches. Nevertheless, data comparing the efficacy of various methods are still unavailable; therefore, no standardized method has been defined to date. Commercialized devices have been developed to improve SVF separation process. Devices offering enzymatic methods are generally more efficient but more expensive than those using mechanical methods. For the widespread use of ADSCs in regenerative therapy, further experiments are essential to describe the standardized isolation procedure with efficacy and cost‐effectiveness.

Long‐term ADSC storage is another barrier to the clinical application of ADSC‐based therapies. Dimethyl sulfoxide remains the most common cryoprotective agent, and xenogeneic serum‐free media has been formulated to avoid immunological reactions and infections.^[^ ^84^ ^]^ Few studies have investigated the optimal temperature and storage duration for ADSCs. Roato et al. demonstrated that ADSCs obtained from cryopreserved adipose tissue at ‐80 °C and ‐196 °C both exhibited viability and differentiation ability comparable to fresh samples.^[^ ^85^ ^]^ In addition, a recent study reported that adipose tissue and ADSCs can be cryopreserved for up to 44 months before use.^[^ ^86^ ^]^ However, the quantity and quality of ADSCs after freeze‐thaw cycles are unpredictable, and the feasibility of quality‐controlled ADSCs storage remains unclear. Cryopreservation of ADSCs remains a great challenge and further investigation is clearly warranted. The long‐term storage of umbilical cord blood has become common because of the establishment of standard procedures. Similarly, a standardized cryopreservation process is essential for constructing ADSCs banks, which will greatly facilitate the clinical application of ADSCs.

## 4 ADSCs in Regenerative Therapies: An Overview of Recent Advancements

### 4.1 ADSCs for Fat Grafting

Owing to its inherent advantages of being the most natural filler and autologous tissue, fat grafting has been extensively used in plastic surgery for various diseases.^[^ ^87^ ^]^ However, inconsistent vascularization and unpredictable survival volume place limitations on the repair size.^[^ ^88^ ^]^ In 2006, Matsumoto et al. first reported a technique named cell‐assisted lipotransfer (CAL).^[^ ^89^ ^]^ They combined aspirated fat with ADSCs to create stem cell‐rich fat grafts and transplanted them subcutaneously into mice with severe combined immunodeficiency. Compared to conventional fat grafting, CAL showed a significantly higher survival rate of transplanted fat with enhanced angiogenesis. Since then, ADSC‐based CAL has been developed and used for facial rejuvenation and cosmetic breast augmentation.^[^ ^90^ ^]^

**Figure 5:**
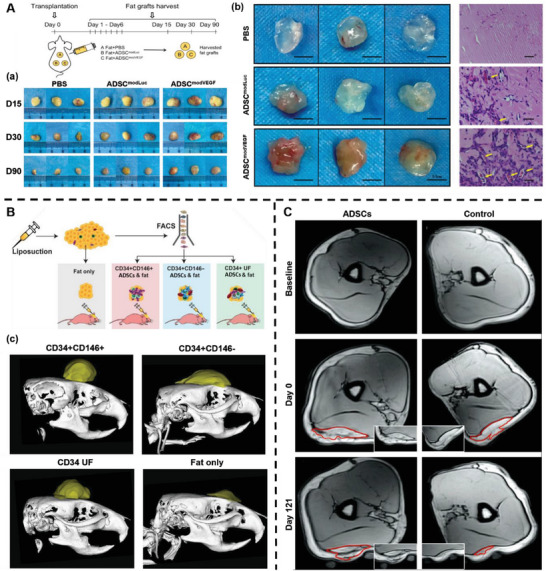
Adipose‐derived stem cells (ADSCs) increase the survival rate of fat grafts. A) Schematic illustration of the co‐transplantation of fat grafts with phosphate‐buffered saline (PBS), ADSCs transfected with Luciferase (ADSC^modLuc^) and ADSCs transfected with VEGF modRNA (ADSC^modVEGF^). (a) Representative gross morphological images of fat grafts at 15, 30 and 90 d following transplantation. (b) Representative gross morphological assessment and hematoxylin and eosin (H&E) staining of extracted fat grafts at 1‐week post‐implantation. Yellow arrows indicate new blood vessel formation within the fat grafts. Reproduced under terms of the CC‐BY license.^[^ ^92^ ^]^ Copyright 2020, The Authors, Published by Springer Nature. B) Schematic illustration of isolation of CD34^+^CD146^+^, CD34^+^CD146−, and CD34^+^ unfractionated (UF) ADSCs by fluorescence‐activated cell sorting (FACS). (c) Micro‐computed tomography of fat grafts at 8 weeks post‐transplantation. Reproduced under terms of the CC‐BY license.^[^ ^93^ ^]^ Copyright 2020, Oxford University Press. C) Magnetic resonance imaging (MRI) of the same areas of healthy participants before implantation and day 0 and 121 after implantation. Reproduced with permission.^[^ ^94^ ^]^ Copyright 2013, Elsevier.

Recently, the efficacy and safety of CAL have been investigated in preclinical and clinical studies. Chen et al. suggested that ADSCs can modulate inflammatory and oxidative responses and increase the survival rate of fat grafts via crosstalk between the Nrf2 and TLR4 pathways, further elucidating the mechanism of ADSCs co‐transplanted with adipose tissue.^[^ ^91^ ^]^ Moreover, considering the importance of revascularization for the survival of fat grafts, methods to augment the proangiogenic ability of ADSCs have become a major research interest. Yu et al. transfected modified mRNA (modRNA) encoding VEGF into ADSCs and transplanted them with human fat into a murine model.^[^ ^92^ ^]^ Although the proangiogenic cytokine VEGF is naturally secreted by ADSCs, premature cell death and low levels of endogenous VEGF after transplantation lead to insufficient angiogenesis. Through VEGF modRNA transfection, ADSCs enhanced their proangiogenic ability and promoted long‐term fat graft survival in vivo (**Figure** **5**A). Similarly, Borrelli et al. isolated a CD34^+^CD146^+^ ADSCs subpopulation by fluorescence‐activated cell sorting. After transplantation, CD34^+^CD146^+^ ADSCs improved the survival rate of fat grafts with increased expression of proangiogenic factors, including VEGF, fibroblast growth factor (FGF), and angiopoietin‐1 (Figure 5B).^[^ ^93^ ^]^ To investigate the therapeutic effect of ADSCs in the clinic, Kølle et al. conducted a clinical trial involving 10 healthy participants.^[^ ^94^ ^]^ They enriched fat grafts with autologous ADSCs in vitro. Then ADSC‐enriched fat grafts and fat grafts without ADSCs enrichment were subcutaneously transplanted into the upper arm. During the 121 day follow‐up, the ADSC‐enriched fat grafts showed significantly higher fat survival than the control grafts, without causing serious adverse events (Figure 5C).

Although discussions about the potential tumorigenicity of ADSCs are ongoing, no clinical trials have indicated that ADSCs increase the risk of oncological recurrence. In a recent clinical trial involving 169 patients after breast cancer surgery, 41 patients received CAL and 64 patients received standard fat grafting, whereas the others did not undergo any fat grafting procedure.^[^ ^95^ ^]^ During at least 5 years of follow‐up, neither standard fat grafting nor CAL increased the oncological recurrence, demonstrating the safety of CAL. Currently, the conclusive guidelines for the use of CAL in breast reconstruction after oncological mastectomy have not yet been formulated. A larger prospective, randomized, multicenter clinical study is required to evaluate the safety of ADSCs transplantation in cancerous environments.

### 4.2 ADSCs for Wound Healing

As a protective shield for the human body, the skin exhibits self‐healing and renewal functions when suffering acute or chronic injuries. Wound healing is a dynamic and complex process involving blood coagulation, inflammation, cell proliferation, and ECM remodeling.^[^ ^96^ ^]^ However, the wound healing process may be compromised under particular circumstances, such as diabetes mellitus or deep burns.^[^ ^97^ ^]^ For refractory wounds which incur clinical and socioeconomic costs, the therapeutic potential of ADSCs has been identified as a promising research direction.

ADSCs are involved in the healing process through their differentiation into skin cells and paracrine effects. Studies have demonstrated that ADSCs can migrate to wound sites and differentiate toward endothelial cells, dermal fibroblasts, and keratinocytes.^[^ ^98^ ^]^ Stromal cell‐derived factor‐1 (SDF‐1) has been proven to be the most highly expressed protein involved in human skin cell migration, which is overexpressed in ADSCs and plays multiple physiological roles via the SDF‐1/CXCR4/CXCR7 axis.^[^ ^99^ ^]^ Interestingly, ADSCs can promote wound healing without homing to the wound bed. Kim et al. compared three different modes of ADSCs administration: topical application, intravenous injection, and intramuscular injection.^[^ ^100^ ^]^ Their results suggested that ADSCs accelerated wound repair independent of their administration techniques. Similarly, Kallmeyer et al. used a rat skin defect model with ADSCs administered systemically or locally. Although ADSCs administered via intravenous injection were rarely detected within the wound bed, they promoted wound healing, possibly through the paracrine pathway.^[^ ^101^ ^]^

**Figure 6:**
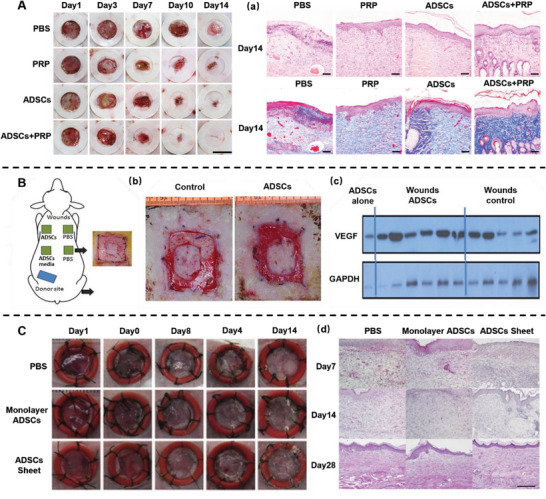
Adipose‐derived stem cells (ADSCs) promote wound healing. A) Representative images of the wounds with phosphate‐buffered saline (PBS), platelet‐rich plasma (PRP), ADSCs and ADSCs+PRP treatment. (a) Hematoxylin and eosin (H&E) staining on day 14 following surgery. Reproduced under terms of the CC‐BY license.^[^ ^102^ ^]^ Copyright 2021, The Authors, Published by Springer Nature. B) Schematic illustration of an ovine burn wound model. (b) Representative images of the wounds on day 15. (c) Western blot staining for vascular endothelial growth factor (VEGF). Reproduced with permission.^[^ ^105^ ^]^ Copyright 2020, Oxford University Press. C) Representative images of the wounds with PBS, monolayer ADSCs and ADSCs sheet treatment. (d) H&E staining of the wound tissue. Reproduced with permission.^[^ ^106^ ^]^ Copyright 2018, Elsevier.

The use of ADSCs in wound healing has yielded encouraging results. To investigate the synergistic effect of ADSCs and platelet‐rich plasma (PRP), Ni et al. subcutaneously injected ADSCs, PRP, PBS, and ADSCs+PRP into rats with full‐thickness skin defects.^[^ ^102^ ^]^ They found that ADSCs combined with PRP induced a higher wound closure rate with a thicker epidermis and an increased number of appendages in the dermis (**Figure** **6**A). In another study, ADSCs were preconditioned with photobiomodulation (PBM) and grafted onto diabetic wounds.^[^ ^103^ ^]^ Their data showed that ADSCs preconditioned with PBM significantly facilitated wound healing both in vitro and in vivo. PBM can modulate hypoxia‐inducible factor‐1*α* (HIF‐1*α*) expression and promote angiogenesis.^[^ ^104^ ^]^ Hence, this pre‐processing method serves as an ideal strategy for overcoming the diabetic‐related impairments in ADSCs. Fujiwara et al. treated full‐thickness burn wounds in an ovine burn model using ADSCs.^[^ ^105^ ^]^ There is an increased demand for oxygen and nutrients during the process of wound healing, but burn injuries generally cause extensive damage to blood vessels. The topical application of ADSCs promoted wound bed blood flow with higher VEGF expression, thus ameliorating burn wound healing (Figure 6B). Yu et al. investigated the function of ADSC sheets in wound healing.^[^ ^106^ ^]^ Compared to monolayer ADSCs, applying ADSCs in a cell sheet format improves cell survival and increases C1q/TNF‐related protein‐3 secretion, which can suppress the recruitment of macrophages to the wound site. When applied to healing‐impaired wounds, ADSCs sheets promoted wound healing with reduced scar formation, thereby representing an effective treatment modality (Figure 6C).

Multiple clinical trials have examined the efficacy and safety of ADSCs for wound healing. Moon et al. harvested ADSCs from the subcutaneous fat tissue of healthy donors and fabricated a hydrogel sheet containing allogeneic ADSCs.^[^ ^107^ ^]^ Fifty‐nine participants with diabetic foot ulcers were randomly divided into two groups and treated with allogeneic ADSC‐hydrogel complex or polyurethane film. During the 12 week follow‐up, the complete wound closure rate in the ADSCs group was distinctly higher than that in the control group, indicating the promising potential of the allogeneic ADSC‐hydrogel complex for treating diabetic foot ulcers. Recently, Zhou et al. conducted a clinical trial involving 296 patients with skin wounds caused by burns or crush injuries.^[^ ^108^ ^]^ Their results demonstrated that ADSCs increased the granulation tissue coverage rate and promoted wound healing without causing adverse events. ADSC‐based therapies represent a promising strategy for wound healing and deserve further investigation.

### 4.3 ADSCs for Bone Regeneration

Bone regeneration is a sophisticated physiological process that is most commonly observed in fracture healing.^[^ ^109^ ^]^ However, the remodeling process may fail to restore bone integrity when complex clinical conditions cause excessive damage to bone tissues, such as large bone defects caused by heavy trauma, infection, and tumor resection, or when the regenerative process is impaired by increased age, comorbidities, and genetic factors.^[^ ^110^ ^]^ The current gold standard for treating these conditions is autologous or allogeneic bone grafting. Nevertheless, they have several disadvantages, including limited accessibility, donor site morbidity, and availability of appropriate materials. A promising alternative is bone tissue engineering in which ADSCs are directly injected into the fracture site or combined with biomimetic scaffolds for bone regeneration.^[^ ^111^ ^]^

ADSCs can be induced to differentiate the osteogenic lineage using a differentiation media containing dexamethasone, ascorbic acid, and beta‐glycerophosphate.^[^ ^112^ ^]^ Dexamethasone promotes osteogenic differentiation through the Wnt/*β*‐catenin pathway, which upregulates a LIM‐domain protein with 4.5 LIM domains, Runx2, and collagen type I.^[^ ^113^ ^]^ Moreover, dexamethasone regulates Runx2 via activation of the *β*‐catenin‐like molecule TAZ and mitogen‐activated protein kinase phosphatase 1.^[^ ^114^ ^]^ Ascorbic acid ensures that collagen chains form an appropriate helical structure, thereby facilitating osteogenesis by increasing collagen type I secretion.^[^ ^115^ ^]^ Beta‐glycerophosphate provides phosphate for hydroxyapatite formation and induces osteogenic gene expression via the phosphorylation of related kinases.^[^ ^116^ ^]^

Furthermore, the effects of additive supplements such as bone morphogenetic proteins (BMPs),^[^ ^117^ ^]^ plasma,^[^ ^118^ ^]^ selenium,^[^ ^119^ ^]^ vitamin D3,^[^ ^120^ ^]^ and human platelet lysates have been widely evaluated.^[^ ^121^ ^]^ As members of the TGF‐*β* family, BMPs are potent inducers of bone formation. They interact with the MAPK, Wnt, Hedgehog, Notch, Akt/mTOR, and miRNA pathways to regulate bone organogenesis. Recently, Yahiro et al. identified atonal homolog 8, which is directly upregulated by the BMP‐Smad1 axis in osteoblasts.^[^ ^122^ ^]^ Their results revealed that BMPs not only promote bone resorption but also induce atonal homolog 8 to inhibit Runx2 and reduce the Rankl/Opg expression ratio in osteoblasts, thus suppressing osteoclastogenesis and preventing excessive bone resorption mediated by BMPs.

In addition to the aforementioned additive supplements, several mechanical stimuli can facilitate osteogenesis in cultured ADSCs. Virjula et al. used pneumatic cell‐stretching devices to expose ADSCs to cyclic equiaxial stretching in an osteogenic medium.^[^ ^123^ ^]^ Their results indicated that stretching promoted osteogenic differentiation by modifying the cell morphology, focal adhesion formation and mechanical properties. Song et al. found that the osteogenic differentiation of ADSCs on osteon‐mimetic 3D nanofibrous scaffolds was significantly higher than that on 2D surfaces, even without osteogenic supplements.^[^ ^124^ ^]^ They further demonstrated that this phenomenon resulted from the stretched cell morphology on the curved sublayer leading to an increased expression of lamin‐A. Moreover, Prè et al. stimulated ADSCs daily at 30 Hz for 45 min for 28 d. Their data suggested that high‐frequency vibration treatment can promote osteogenic differentiation.^[^ ^125^ ^]^ Similarly, the effectiveness of electrical stimulation,^[^ ^126^ ^]^ electromagnetic fields,^[^ ^127^ ^]^ and flow intensity has been observed in recent years.^[^ ^128^ ^]^ These mechanical stimuli may serve as candidate osteogenic enhancers for ADSCs.

Several preclinical studies have investigated the use of ADSCs in bone regeneration. Zhang et al. engineered ADSCs to overexpress FGF and injected them into fracture sites.^[^ ^129^ ^]^ Their results demonstrated that ADSCs overexpressing FGF accelerated fracture healing by increasing growth factor secretion and facilitating the remodeling of collagen into a mineralized callus. To overcome the impairment of bone healing after osteomyelitis, Wagner et al. administered ADSCs into the bone defect area after sufficient debridement of the infected bones. The authors found that ADSCs can restore bone regeneration via the elevation of osteoblastogenesis and downregulation of B cells and osteoclasts, shedding light on the regenerative capacity of ADSCs in the post‐infectious inflammatory state.^[^ ^130^ ^]^

ADSCs have been combined with various biomaterials to achieve optimal bone regeneration in tissue engineering. For example, a composite of ADSCs and heterogeneous deproteinized bone exhibits strong osteogenic ability and repairs bone defects well.^[^ ^131^ ^]^ Du et al. proposed the utilization of the multi‐lineage differentiation capacity of ADSCs. They induced ADSCs into endothelial cells and assembled them into mesoporous bioactive glass (MBG) scaffolds.^[^ ^132^ ^]^ The prevascularized MBG scaffolds were then combined with osteogenically induced ADSCs. The strategy of time‐phased sequential utilization of ADSCs promoted better bone formation than nonvascularized MBG‐carrying osteogenically induced ADSCs, which may result from the higher survival rate of seeded ADSCs. Recently, stem cell spheroids have shown great potential as functional building blocks for bone tissue engineering. Lee et al. combined ADSCs with PDGF and biomineral coated fibers to form spheroids. The ADSCs spheroids exhibited higher endothelial lineage mRNA expression and osteogenic capability.^[^ ^133^ ^]^ Similarly, in a study proposed by Ahmad et al., ADSCs were assembled with adenosine and polydopamine‐coated fibers to construct spheroids.^[^ ^134^ ^]^ Their data indicated that ADSCs spheroids impregnated with engineered fibers enabled adenosine delivery and promoted bone regeneration with enhanced osteogenic differentiation. In summary, engineered ADSCs spheroids are promising alternatives for vascularized bone regeneration.

**Figure 7:**
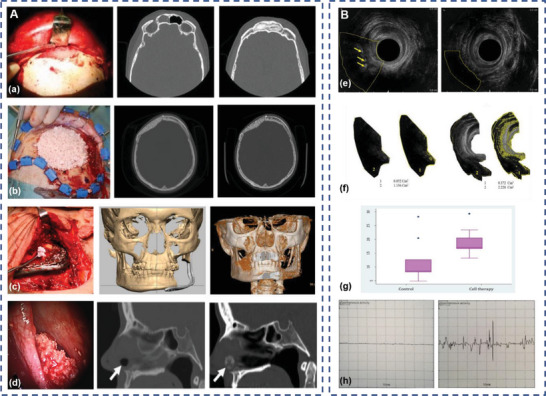
Clinical application of adipose‐derived stem cells (ADSCs) for cranio‐maxillofacial hard‐tissue regeneration and external anal sphincter injury treatment. A) Intraoperative photograph and computed tomography (CT) of frontal sinus regeneration (a), cranial repair (b), mandibular reconstruction (c), and nasal septal repair (d) using ADSCs. Reproduced with permission.^[^ ^135^ ^]^ Copyright 2014, Oxford University Press. B) Images of endorectal sonography (e), the area occupied by the muscle (f), the median percentage of area occupied by the muscle (g) and sample electromyography (h) of the control group (left) and the ADSCs group (right) at 2 months after surgery, arrows indicate fibrous tissue. Reproduced under terms of the CC‐BY license.^[^ ^147^ ^]^ Copyright 2017, The Authors, Published by Springer Nature.

Encouraging results obtained from the preclinical application of ADSCs in bone tissue engineering have prompted several clinical trials. In one study involving 13 patients with craniomaxillofacial hard‐tissue defects, autologous ADSCs were seeded onto either bioactive glass or *β*‐tricalcium phosphate scaffolds and transplanted into the bone defect area.^[^ ^135^ ^]^ According to the follow‐up results, successful bone regeneration was observed in 10 of 13 patients (**Figure** **7**A). One septal perforation case attributed the failure to an uncontrolled nasal picking habit, and two cranial defect cases required reoperation because of sustained bone resorption. In another six year clinical follow‐up study of five cranial defect cases, patients received cranioplasties using ADSCs, beta‐tricalcium phosphate granules, and supporting meshes.^[^ ^136^ ^]^ Promising results have been reported in short‐term follow‐up with increased graft bone density. Nevertheless, the long‐term clinical results are not satisfactory, partially due to the resorption of the graft and partially due to tumor recurrence or late infection. In the field of bone regeneration, the efficacy and safety of ADSC‐based therapies need to be further investigated for wide clinical applications.

### 4.4 ADSCs for Skeletal Muscle Repair

Despite the innate regenerative capacity of skeletal muscles, severe muscle defects may exceed their inherent repair properties, leading to permanent functional impairment.^[^ ^137^ ^]^ Volumetric muscle loss, which is characterized by the critical loss of skeletal muscle tissues, may result from traumatic injuries, tumor excision, or muscular dystrophy.^[^ ^138^ ^]^ Current treatments involving autologous muscle flaps or grafts are limited in their capacity to provide a functional reconstruction of the injured muscle tissue.^[^ ^139^ ^]^ The management of skeletal muscle injuries requires more effective approaches for muscle regeneration and ultimate functional recovery. Therefore, skeletal muscle tissue engineering aimed at neuromuscular recovery may be an attractive option for volumetric muscle loss therapy.

Myogenic differentiation of ADSCs has been observed in myogenic medium, as demonstrated by the expression of the muscle‐specific markers MyoD and myosin heavy chain.^[^ ^140^ ^]^ Moreover, ADSCs exhibited higher myogenic potential when pretreated with IL‐4 and SDF‐1.^[^ ^141^ ^]^ To compare the effects of ADSCs and BM‐MSCs on skeletal muscle injury. Moussa et al. intramuscularly injected ADSCs or BM‐MSCs into rats with lacerated gluteal muscles.^[^ ^142^ ^]^ They found that both ADSCs and BM‐MSCs improved the healing process of skeletal muscle lacerations, with less fibrosis observed in the ADSC‐treated group. This study revealed that ADSCs may have a higher therapeutic potential than BM‐MSCs in treating skeletal muscle injury and could be a more promising alternative in skeletal muscle tissue engineering.

To explore the effect of ADSCs on muscle repair, Liu et al. injected ADSCs and HIF‐1*α*‐silenced ADSCs into a mouse model of muscle ischemia. Their results demonstrated that ADSCs promoted ischemic muscle regeneration by inducing M2 macrophage polarization via the HIF‐1*α*/IL‐10 pathway, while the therapeutic effect decreased in HIF‐1*α*‐silenced ADSCs.^[^ ^143^ ^]^ In addition, Gorecka et al. implanted ADSCs into the crushed muscles of mice and tracked them using optical projection tomography.^[^ ^144^ ^]^ The authors found that ADSCs promoted skeletal muscle regeneration without directly participating in muscle fiber formation. Hence, they suggested that ADSCs could enhance skeletal muscle repair via direct intercellular communication or paracrine pathways.

Recently, the combination of ADSC‐based therapies and other treatments has been proposed as an effective strategy for skeletal muscle repair. For example, Yin et al. investigated a combined therapy using ADSCs and extracorporeal shock waves (ECSW) for muscle injury. In this study, rats with limb ischemia‐reperfusion injury received ADSCs injections and ECSW.^[^ ^145^ ^]^ Their results indicated that ECSW‐ADSC treatment appeared to be more effective than either treatment alone in improving muscle ischemia‐reperfusion injury. Likewise, in a study conducted by Sarveazad et al., rabbits that underwent sphincterotomy received a combination of ADSCs and low‐level laser therapy.^[^ ^146^ ^]^ The authors found that laser‐ADSC treatment was superior to either treatment alone in enhancing myogenesis, angiogenesis, and functional recovery after anal sphincter injury. To confirm the efficacy of ADSCs in treating external anal sphincter injury, Sarveazad et al. conducted another clinical trial. They divided 18 patients with sphincter defects into two groups: nine patients received ADSCs injection during repair surgery treatment, while the others received repair surgery alone.^[^ ^147^ ^]^ At 2 months after surgery, the ratio of the muscle tissue area to total lesion area in the ADSCs group was significantly higher than that in the control group. The decrease of fibrous tissue and electromyography results indicated improved contractile function, which plays a key role in long‐term curative effects (Figure 7B). However, the external anal sphincter injury in the patients resulted from different causes, such as trauma or a high‐sphincter fistula. Considering the limited sample size and short follow‐up time, the heterogeneity of patients may lead to inaccurate results. Clinical trials with larger sample sizes and long‐term follow‐up may be a major step forward in this field.

### 4.5 ADSCs for Tendon Reconstruction

The tendon is a highly organized collagenous connective tissue that ensures force transmission between the muscles and bones.^[^ ^148^ ^]^ Tendinopathy refers to a pathology of the tendon arising from repetitive loading, which is characterized by abnormalities in the composition and microstructure of the tendon.^[^ ^149^ ^]^ Studies have reported pain, functional decline, and reduced quality of life in patients with tendinopathy.^[^ ^150^ ^]^ Nevertheless, tendinopathy treatment remains challenging. Owing to the low cellularity and limited vascularity of the tendon tissue, repaired tendon tissue rarely attains pre‐injury function, leading to degenerative changes and a high risk of re‐injury.^[^ ^151^ ^]^ Given the limited success of the current clinical treatments, tendon tissue engineering is expected to be an alternative therapeutic approach.

At present, there is no standard induction protocol for the tenogenic differentiation of ADSCs, whereas various strategies have been proposed with encouraging results. Rao et al. prepared a soluble, DNA‐free, bovine tendon‐derived ECM (tECM) using a urea‐based method.^[^ ^152^ ^]^ The tECM retains collagen and many other noncollagenous tendon ECM components. When treated with the tECM, ADSCs showed enhanced proliferation and tenogenic differentiation, demonstrating the strong pro‐tenogenic bioactivity of the tECM. In addition, Guo et al. combined topographical and mechanical cues to facilitate the tenogenic differentiation of ADSCs. They developed a mechanoactive fibrous scaffold with shape memory capability that can exert an in situ mechanical stimulus on ADSCs and induce cellular elongation along the direction of fiber alignment.^[^ ^153^ ^]^ This mechanoactive fibrous substrate may improve the efficacy of tendon reconstruction by enhancing the tenogenic differentiation of ADSCs.

**Figure 8:**
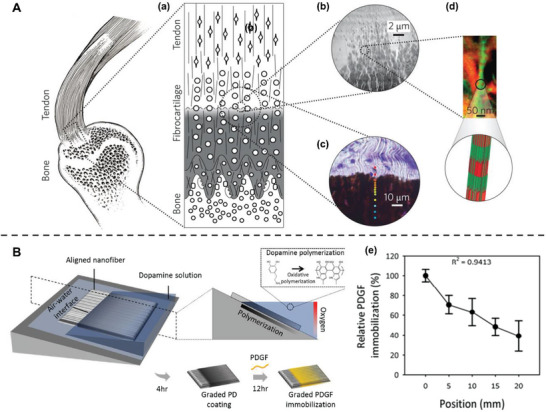
Spatially control of adipose‐derived stem cells (ADSCs) differentiation mimicking tendon‐to‐bone attachment. A) The hierarchical structure of the tendon‐to‐bone attachment. (a) Schematic of transitional tissue. (b) Transmission electron microscopy (TEM) of the mineral gradient. (c) Raman microprobe results of mineral content. Color dots indicating mineral content (red, low; blue, high). (d) TEM‐electron energy loss spectroscopy image (red, mineral; green, tropocollagen). Reproduced with permission.^[^ ^154^ ^]^ Copyright 2017, Springer Nature. B) Fabrication of a platelet‐derived growth factor (PDGF) gradient aligned nanofiber surface by controlling oxidative polymerization of dopamine. (e) The relative percentage of immobilized PDGF at different positions on a PDGF gradient nanofiber. Reproduced with permission.^[^ ^155^ ^]^ Copyright 2018, Elsevier.

Recently, a strategy combining topographical and biological cues has also been proven effective, especially in tendon‐to‐bone attachment regeneration. The tendon‐to‐bone attachment is a highly specialized transitional tissue with smooth gradients in the mineralization and alignment of collagen fibers (**Figure** **8**A).^[^ ^154^ ^]^ Hence, tendon‐to‐bone tissue engineering requires the combined effects of various stimuli to achieve optimal tissue regeneration. Based on this theory, Perikamana et al. immobilized PDGF on aligned electrospun nanofibers using polydopamine chemistry.^[^ ^155^ ^]^ The growth factor and scaffold morphology showed a synergistic effect, and exhibited the maximum tenogenic differentiation capability of ADSCs. Notably, PDGF immobilized in a gradient spatially controlled the phenotypic differentiation of ADSCs, mimicking the tendon‐to‐bone insertion site (Figure 8B). Similarly, Calejo et al. combined platelet lysates with an electrospun fiber core to fabricate graded 3D scaffolds that were able to release biological factors continuously in physiological or inflammatory settings.^[^ ^156^ ^]^ The 3D functional scaffolds with gradients in composition and topography can not only promote ADSCs proliferation, but also regulate the differentiation of ADSCs for enthesis regeneration. Based on the aforementioned results, a strategy combining topographical and biological cues can be a potential tool for ADSC‐based tendon reconstruction.

To investigate the efficacy of ADSCs in tendon regeneration in vivo, Norelli et al. injected tenogenically differentiated ADSCs, undifferentiated ADSCs, and hydrogels into a rat Achilles excision defect model.^[^ ^157^ ^]^ The authors found that ADSCs improved the biomechanical properties of repaired tendons more than hydrogel alone, while the collagen fiber dispersion range closest to the normal tendon was observed in the group treated with tenogenically differentiated ADSCs. In addition, ADSCs sheets have been demonstrated to be a feasible cell delivery tool for tendon reconstruction. Shin et al. fabricated the ADSCs sheets with a temperature‐responsive dish and transplanted them into rats undergoing procedures to create rotator cuff tears.^[^ ^158^ ^]^ Their data indicated that the ADSCs sheets significantly enhanced the biomechanical properties of the repaired rotator cuff. In another study, ADSCs sheets stimulated by growth differentiation factor 5 (GDF‐5) were combined with nanoyarn scaffolds and implanted into the patellar tendon defect area of rabbits.^[^ ^159^ ^]^ The GDF‐5‐induced ADSCs sheets expressed higher levels of tenogenesis‐related markers and promoted functional tendon regeneration, indicating a broad application prospects of ADSCs sheets.

Regarding the clinical trials, Kim et al. evaluated the effect of autologous ADSCs injection on the clinical outcomes of rotator cuff tear. In their study, 35 patients underwent arthroscopic rotator cuff repair with an injection of autologous ADSCs loaded in fibrin glue, whereas 35 patients underwent repair surgery alone.^[^ ^160^ ^]^ The results suggested that the ADSCs injection during rotator cuff repair could significantly decrease the retear rate, whereas the function of the repaired tissue was similarly ameliorated in both groups. Recently, Randelli et al. conducted a clinical trial that included 44 patients with degenerative posterosuperior rotator cuff tears. The patients were randomly divided into two groups and underwent arthroscopic rotator cuff repair, followed by the injection of autologous microfragmented lipoaspirate tissue.^[^ ^161^ ^]^ During the 24‐month follow‐up, microfragmented lipoaspirate tissue containing ADSCs effectively promoted the functional rotator cuff repair. Although still in the early stage of clinical application, intraoperative ADSCs injections are expected to provide a suitable microenvironment for tendon regeneration and improve the clinical outcomes of rotator cuff tears.

### 4.6 ADSCs for Cartilage Regeneration

The progression of cartilage defects may lead to osteoarthritis or even arthroplasty due to the poor intrinsic regenerative capacity of human cartilage tissue.^[^ ^162^ ^]^ Current clinical methods are deficient because they form fibrocartilage instead of hyaline cartilage, which is associated with joint dysfunction and long‐term sequelae.^[^ ^163^ ^]^ The management of cartilage defects remains one of the most intractable clinical problems around the world. Recently, cartilage tissue engineering has become a major research interest in the treatment of articular cartilage defects and the prevention of osteoarthritis progression.^[^ ^164^ ^]^

ADSCs harvested from the infrapatellar fat pad (IFP) have been demonstrated to show higher chondrogenic potential than those isolated from subcutaneous fat (SCF).^[^ ^165^ ^]^ Garcia et al. tested the chondrogenesis of donor‐matched chondrocytes and ADSCs isolated from IFP and SCF. Their results indicated that chondrocytes and IFP‐derived ADSCs generated significantly more glycosaminoglycans than SCF‐derived ADSCs, with some notable variation between donors.^[^ ^166^ ^]^ The authors also confirmed that Sox‐9, the master regulator of chondrogenesis, was highly expressed in IFP‐derived ADSCs and correlated positively with the expression of Collagen II and ACAN, at least partially explaining the potent chondrogenic potential of IFP‐derived ADSCs. In addition, ADSCs are likely to be positively affected by various inducer molecules, including TGF‐*β*, BMPs, and FGF.^[^ ^167^ ^]^ These factors can be supplemented to the culture medium alone or in combination to promote the chondrogenesis of ADSCs. Liao et al. evaluated the synergistic effects of Sox‐9 and BMP‐2 on MSC differentiation.^[^ ^168^ ^]^ Their data indicated that the overexpression of Sox‐9 potentiates the chondrogenic differentiation and inhibits the osteogenic differentiation of MSCs induced by BMP‐2.

**Figure 9:**
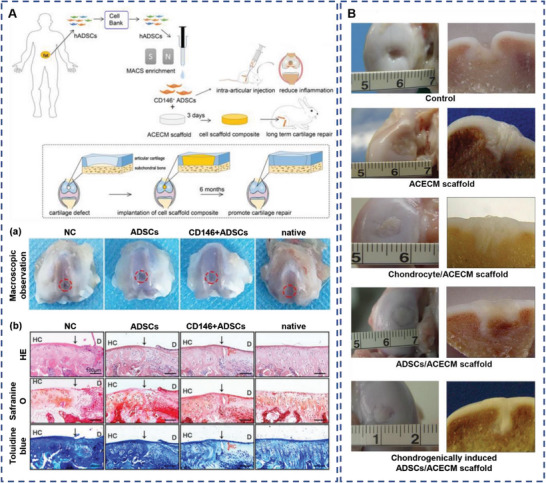
Adipose‐derived stem cells (ADSCs) combined with articular cartilage extracellular matrix (ACECM) promote cartilage regeneration. A) Overview of experimental design. (a) Representative images of defects treated with phosphate‐buffered saline (PBS) (negative control), ADSCs, CD146^+^ ADSCs, and the sham‐operated group at 2 weeks following surgery. (b) Histological analysis of the defected area using hematoxylin and eosin (H&E), safranin O, and toluidine blue. Black solid arrows denote the repair interface. Red solid arrows denote the depth of the repaired cartilage. HC, host cartilage; D, defect area; RC, repaired cartilage. Reproduced under terms of the CC‐BY license.^[^ ^175^ ^]^ Copyright 2022, Ivyspring International Publisher. B) General joint observations (left) and longitudinal section (right) of the cartilage regeneration at 3 months after surgery. Reproduced with permission.^[^ ^176^ ^]^ Copyright 2020, John Wiley and Sons.

As carriers for cells to maintain and support crucial characteristics, biomaterials for tissue engineering should induce cell adhesion and provide a favorable microenvironment for cell proliferation and differentiation.^[^ ^169^ ^]^ Hydrogel materials are characterized by high water content, which is similar to native cartilage tissue. Injectable hydrogels have been widely used in cartilage regeneration because of their good biocompatibility, and the encapsulated cells can be injected into defective areas of any shape.^[^ ^170^ ^]^ For example, ADSCs combined with glycol chitosan/dibenzaldehyde‐terminated polyethylene glycol (GCS/DF‐PEG) hydrogels have been demonstrated to facilitate obvious cartilage regeneration in vivo.^[^ ^171^ ^]^ Likewise, Bhattacharjee et al. fabricated an injectable amnion membrane (AM) hydrogel that could self‐assemble in situ and hold ADSCs at the target site.^[^ ^172^ ^]^ In this study, ADSCs and AM hydrogels exhibited synergistic anti‐inflammatory and chondroprotective effects, demonstrating the therapeutic potential of the ADSCs and AM hydrogel complexes for cartilage tissue engineering. Additionally, Cho et al. constructed a dual‐delivery system of ADSCs and IGF‐1 in coacervate‐embedded composite hydrogels.^[^ ^173^ ^]^ Owing to the protective effect of coacervates on chondrogenic factor IGF‐1, the dual‐delivery platform could induce chondrogenic differentiation of embedded ADSCs and effectively promote cartilage regeneration. The articular cartilage extracellular matrix (ACECM) possesses chondroinductive property and serves as the best biomaterial for mimicking native cartilage.^[^ ^174^ ^]^ Studies have demonstrated the feasibility of this biomaterial for ADSC‐based cartilage regeneration because this raw material of cartilage can provide a proper microenvironment in vivo. Li et al. sorted a CD146^+^ ADSCs subpopulation using magnetic activated cell sorting (MACS), which enhanced pluripotency and self‐renewal potential.^[^ ^175^ ^]^ After intra‐articular injection into a rat osteochondral defect model, CD146^+^ ADSCs exhibited improved inflammation‐modulating property. The authors then combined them with ACECM and transplanted the cell scaffold to rabbit cartilage defects. The CD146^+^ ADSC‐ACECM composites produced less subcutaneous inflammation, and promoted better long‐term cartilage regeneration (**Figure** **9**A). Likewise, another study by Lu et al. proved the effectiveness of ADSC‐ACECM composites for repairing cartilage defects, showing the promising prospects of this tissue‐engineered biomaterial (Figure 9B).^[^ ^176^ ^]^

A clinical trial conducted by Lee et al. evaluated the efficacy and safety of intra‐articular ADSC injections for the treatment of osteoarthritis. Autologous ADSCs were administered intra‐articularly to 12 patients with knee osteoarthritis.^[^ ^177^ ^]^ During the 6‐month follow‐up, ADSC injections provided significant improvements in function and pain levels without causing adverse events. In one dose‐escalation trial designed by Pers et al.,18 patients with osteoarthritis were divided into three groups, which received ADSC injections at low dose (2 × 10^6^), medium dose (10 × 10^6^), and high dose (50 × 10^6^), respectively.^[^ ^178^ ^]^ Functional improvement and pain relief were observed in all treatment groups, whereas statistical significance was identified only in patients treated with a low dose. Except for the limited sample size, the inverse dose effect of ADSC injection may result from the stimulatory effect of higher levels of inflammation on ADSCs, as reflected by the highest initial pain level in the lowest dose group. This hypothesis emphasizes the relationship between the therapeutic effects of ADSCs and initial disease activity. Further studies of the effect of ADSCs injection on osteoarthritis in future clinical trials with larger sample sizes and long‐term follow‐up are required.

### 4.7 ADSCs for Cardiac Repair

Cardiovascular disease (CVD) is the leading cause of death, morbidity, and disability worldwide.^[^ ^179^ ^]^ Ischemic heart disease, specifically myocardial infarction (MI), is a type of CVD caused by the erosion of the coronary artery endothelium or disruption of vulnerable atherosclerotic plaques.^[^ ^180^ ^]^ Although the mortality following MI has been substantially reduced in recent years, considerable opportunities for improvement remain, such as mitigating reperfusion injury, ameliorating adverse remodeling, and inducing cardiac regeneration.^[^ ^181^ ^]^ Stem cell therapy could further ameliorate the clinical outcome of MI, and ADSC therapy has been widely investigated as a promising strategy.

The spontaneous cardiomyogenic differentiation of ADSCs was first observed in semisolid methylcellulose medium, where cardiomyocyte‐like cells expressed specific cardiac markers and displayed a pacemaker activity.^[^ ^182^ ^]^ Since then, various methods have been explored for the cardiomyogenic differentiation of ADSCs. To increase the sensitivity of ADSCs to TGF‐*β*1, Zhang et al. used a polyethylene glycol‐conjugated phospholipid derivative (DMPE‐PEG) as a scaffold to bind recombinant TGF‐*β*1 receptor I to the surface of ADSCs.^[^ ^183^ ^]^ Their results showed that the cardiomyogenic differentiation of ADSCs was significantly promoted by enhancing Smad2/3 phosphorylation. In addition, genetic modification of ADSCs has offered a potential route for inducing their differentiation into cardiomyocytes. Narita et al. directly reprogrammed ADSCs using six transcription factors (Baf60c, Gata4, Gata6, Klf15, Mef2a, and Myocd).^[^ ^184^ ^]^ These transcription factors can induce the differentiation of ADSCs into cardiac cells. When implanted into the infarct border zone, the reprogrammed ADSCs exhibited a higher survival rate and significantly reduced the infarction scar area, which may prevent left ventricular remodeling and cardiac functional impairment.

**Figure 10:**
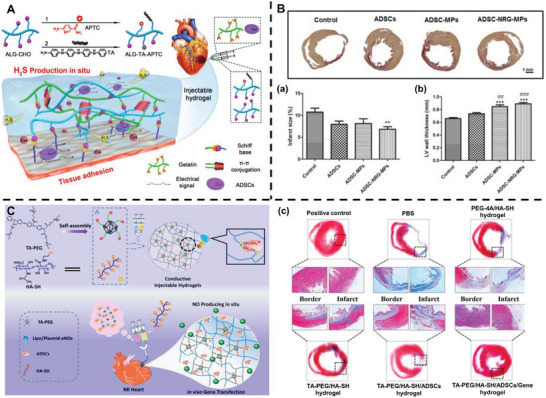
Adipose‐derived stem cells (ADSCs) facilitate cardiac repair. A) Schematic illustration of the ADSCs‐loaded conductive hydrogen sulfide‐releasing hydrogel. Reproduced with permission.^[^ ^185^ ^]^ Copyright 2019, American Chemical Society. B) Sirius red staining of the infarcted hearts at 3 months after different treatments. Quantification of infarct size (a) and the left ventricular (LV) wall thickness (b). Reproduced with permission.^[^ ^186^ ^]^ Copyright 2017, Elsevier. C) Schematic illustration of the construction of an injectable conductive hydrogel encapsulating plasmid DNA‐eNOs and ADSCs. (c) Masson's trichrome staining of cardiac structures. Reproduced with permission.^[^ ^187^ ^]^ Copyright 2018, Elsevier.

ADSCs assembled with various functional biomaterials are effective for cardiac repair in several preclinical studies. Liang et al. fabricated an ADSCs‐loaded conductive hydrogen sulfide‐releasing hydrogel that mimicked the slow and continuous release of endogenous sulfides.^[^ ^185^ ^]^ Owing to its anti‐inflammatory and proangiogenic properties, hydrogen sulfide ameliorated the harsh microenvironment, showing a synergistic effect with ADSCs in MI treatment (**Figure** **10**A). Moreover, Díaz‐Herráez et al. combined ADSCs with microparticles (MPs) loaded with neuregulin (NRG). The adhesion of ADSCs to NRG‐MPs led to improved cell engraftment and neoangiogenesis, favoring synergy for inducing overall cardiac remodeling (Figure 10B).^[^ ^186^ ^]^ Similarly, Wang et al. constructed an injectable conductive hydrogel through the self‐assembly of tetraaniline‐polyethylene glycol diacrylate (TA‐PEG) and thiolated hyaluronic acid (HA‐SH).^[^ ^187^ ^]^ Subsequently, ADSCs and plasmid DNA encoding endothelial nitric oxide synthase (eNOs) were loaded onto the hydrogel. After myocardial injection of the TA‐PEG/HA‐SH/ADSCs/Gene hydrogel, upregulated expression of eNOs was observed, accompanied by reduced infarction size, less fibrotic area, enhanced neoangiogenesis and increased ejection fraction (Figure 10C). These studies verified the efficacy of ADSCs in improving cardiac function and suggested that the combined approach is a robust therapeutic strategy for myocardial repair.

The poor survival of engrafted ADSCs in an ischemic environment impairs their therapeutic efficacy for cardiac repair after myocardial infarction. In addition to engineered biomaterials, pharmacological treatment with ADSCs can promote the retention of transplanted cells.^[^ ^185^, ^186^, ^187^ ^]^ For instance, rosuvastatin enhances the functional survival of ADSCs in MI treatment via the PI3K/Akt and MEK/ERK pathways.^[^ ^188^ ^]^ Recently, Yan et al. identified C1q/tumor necrosis factor‐related protein‐9 (CTRP9) as a novel cardiokine required for a healthy microenvironment that facilitates ADSCs engraftment in MI tissue.^[^ ^189^ ^]^ CTRP9 promotes ADSCs survival via N‐cadherin/ERK/Nrf2 signaling, stimulates ADSCs migration via N‐cadherin/ERK/MMP‐9 signaling, and attenuates cardiomyocyte death by upregulating antioxidative proteins. These results suggested that CTRP9 may optimize the cardioprotective effects of ADSCs and enhance their therapeutic efficacy.

Clinical trials of ADSCs for myocardial repair are ongoing. Kastrup et al. injected allogeneic ADSCs into the infarct border zone in 10 patients with ischemic heart failure.^[^ ^190^ ^]^ During the 6 month follow‐up, they found that the left ventricular ejection fraction and exercise capacity increased without adverse events, demonstrating the efficacy and safety of ADSCs injection. Similarly, in another clinical trial conducted by Qayyum et al., 40 patients with refractory angina received intramyocardial injections of autologous ADSCs, whereas 20 others received saline injections.^[^ ^191^ ^]^ Compared to the placebo group, the injection of ADSCs improved cardiac symptoms, whereas exercise capacity remained unchanged. More clinical trials of ADSC transplantation for myocardial restoration are expected in the near future.

### 4.8 ADSCs for Nerve Regeneration

Peripheral nerve injuries due to trauma or surgical complications can cause demyelination and subsequent degeneration of the distal stump.^[^ ^192^ ^]^ In the presence of a nerve gap where tension‐free neurorrhaphy is impossible, autologous nerve grafting remains the gold standard for bridging the large defect.^[^ ^193^ ^]^ However, the application of nerve grafting is limited by several disadvantages, including finite graft supply, donor site morbidity, and different nerve structures involved in peripheral nerve injuries.^[^ ^194^ ^]^ Nerve regeneration has emerged as a potential solution to these limitations, which not only bridges the gap but also preserves the biological properties of the specific nerve type to obtain an optimum clinical outcome.^[^ ^195^ ^]^ ADSCs have been proposed as a promising option for nerve regeneration owing to their potential to differentiate into the neural lineage and easy accessibility.^[^ ^196^ ^]^

Although the efficiency of traditional methods to induce the neurogenic differentiation of ADSCs remains unsatisfactory, various novel strategies have recently been explored.^[^ ^197^ ^]^ Sun et al. suggested an intermittent induction method with the alternate use of complete and incomplete induction media.^[^ ^198^ ^]^ Their results showed that ADSCs induced using this method could differentiate into Schwann cell‐like cells and repair sciatic nerve defects in rats more effectively than those induced using traditional methods. Moreover, Wu et al. found that the combination of electrical stimulation and chemical induction presented a synergistic effect on accelerating the differentiation and maturation of ADSCs toward Schwann cell‐like cells by upregulating the expression of myelination‐related gene markers and promoting growth factor secretion.^[^ ^199^ ^]^ Huang et al. demonstrated that fibroblast growth factor 9 (FGF9) can induce the functional differentiation of ADSCs into Schwann cells via the FGF9‐FGFR2‐Akt pathway.^[^ ^200^ ^]^ When transplanted into a rat sciatic nerve injury model, the FGF9‐induced ADSCs participated in myelin sheath formation and facilitated axonal regrowth to promote nerve regeneration.

**Figure 11:**
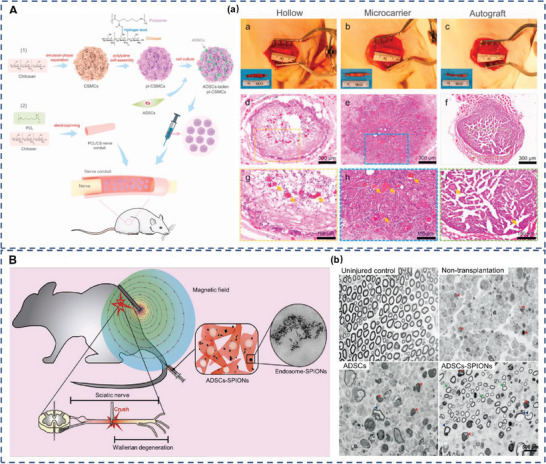
Adipose‐derived stem cells (ADSCs) promote peripheral nerve regeneration. A) Schematic of the preparation process of the ADSCs‐laden polylysine‐decorated macroporous chitosan microcarriers (pl‐CSMCs) and their application in nerve repair. (a) Representative images and hematoxylin and eosin (H&E) staining of the regenerated nerve tissue at the midportion of the nerve guide conduits (NGCs) or autograft 12 weeks after implantation. Yellow arrows indicate blood vessels. Reproduced under terms of the CC‐BY license.^[^ ^202^ ^]^ Copyright 2021, The Authors, Published by Elsevier. B) Schematic illustration of magnetic targeted ADSCs therapy. (b) Analysis of semithin sections of axonal bundles in uninjured control sciatic nerve and the distal stump of non‐transplanted, transplanted with ADSCs and ADSCs‐superparamagnetic iron oxide nanoparticles (SPIONs) transplanted group at 7 days post‐injury. Arrows indicate intact axons; arrowheads indicate irregular axons; asterisks indicate myelin and axon debris. Reproduced with permission.^[^ ^203^ ^]^ Copyright 2021, Elsevier.

Several preclinical studies have examined the efficacy of ADSCs in peripheral nerve regeneration. Hsu et al. activated neurotrophic genes *BDNF*, *GDNF* and *NGF* in ADSCs sheets using CRISPR activation system.^[^ ^201^ ^]^ After direct administration, the functionalized ADSCs sheets stimulated axon regeneration, remyelination, and nerve reinnervation in a rat model of sciatic nerve injury. In a study conducted by Sun et al., ADSCs were loaded onto polylysine‐decorated macroporous chitosan microcarriers and injected into nerve guide conduits (NGCs).^[^ ^202^ ^]^ They found that microcarrier‐based ADSCs transplantation effectively ameliorated the regenerative effect of NGCs, indicating a good potential for peripheral nerve regeneration (**Figure** **11**A). Recently, Soto et al. proposed a magnetic targeted ADSCs therapy for nerve regeneration.^[^ ^203^ ^]^ They combined ADSCs with citric acid‐coated superparamagnetic iron oxide nanoparticles and transplanted them into a rat model of sciatic nerve injury. Using magnetic targeting, ADSCs were recruited to the injured site and improved recovery compared to using ADSCs alone (Figure 11B). Despite the encouraging results obtained from these studies, clinical trials using ADSCs for peripheral nerve regeneration are limited. Further evaluation is indispensable for the progression toward clinical applications.

Injury to any part of the spinal cord can cause severance of axons and death of neurons, leading to permanent neurological dysfunction below the site of damage.^[^ ^204^ ^]^ While a therapeutic approach to spinal cord injury repair is unforeseeable, ADSC‐based therapies represent a promising strategy for neuroprotection and nerve regeneration.^[^ ^205^ ^]^ Sarveazad et al. investigated the combined application of ADSCs and low‐level laser.^[^ ^206^ ^]^ Their results demonstrated that the co‐administration of ADSCs and laser ameliorated motor function recovery, hyperalgesia, and allodynia to a greater extent than ADSCs alone, with an increased number of axons around the cavity. Yuan et al. designed a cell‐adaptable neurogenic (CaNeu) hydrogel that alleviated neuroinflammation by inducing M2 polarization of the recruited macrophages.^[^ ^207^ ^]^ The CaNeu‐hydrogel‐mediated ADSCs delivery promoted axonal growth and eventually led to the improved functional repair of spinal cord injury in rats. It is worth mentioning that one patient with a high cervical American Spinal Injury Association Impairment Scale grade A spinal cord injury received an intrathecal injection of autologous ADSCs, 11 months after the injury and 5 months after the plateau of neurologic improvement.^[^ ^208^ ^]^ After 18 months of ADSC treatment, the patient recovered to American Spinal Injury Association Impairment Scale grade C but regrettably continued to be wheelchair‐bound. The meaningful signs of improvement in this case indicated the feasibility of intrathecal ADSCs administration, and the neurological status warrants further clinical evaluation.

### 4.9 Potential Use of ADSCs in Combating COVID‐19

Coronavirus disease 2019 (COVID‐19) is a severe acute respiratory illness caused by a novel coronavirus, severe acute respiratory syndrome coronavirus 2 (SARS‐CoV‐2). Since the emergence of COVID‐19 in late December 2019, it has become a global pandemic of respiratory ailments, imposing a heavy burden on patients and healthcare systems worldwide.^[^ ^209^ ^]^ The viral entry of SARS‐CoV‐2 is mediated by viral spike protein and host angiotensin‐converting enzyme 2 (ACE2) interaction.^[^ ^210^ ^]^ The functions of multiple organs are impaired in COVID‐19 patients, such as the heart, liver, kidney, and digestive organs, while severe respiratory illness is generally the primary outcome because ACE2 is highly expressed in alveolar type II cells and capillary endothelial cells.^[^ ^211^ ^]^ Viral lung infection drives a pro‐inflammatory cytokine storm that results in lung tissue edema, acute respiratory distress, air exchange dysfunction, secondary infection, and even death.^[^ ^212^ ^]^

**Figure 12:**
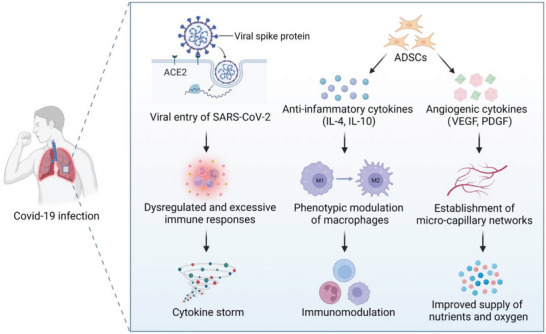
Schematic illustration of adipose‐derived stem cells (ADSCs) for treating coronavirus disease 2019 (COVID‐19). Severe acute respiratory syndrome coronavirus 2 (SARS‐CoV‐2) can enter cells through angiotensin‐converting enzyme 2 (ACE2), thus lung tissues with high expression of ACE2 become the main targets for the novel coronavirus to invade. Dysregulated and excessive immune responses arising from virus infection cause a cytokine storm. ADSCs can secrete anti‐inflammatory cytokines that facilitate the phenotypic modulation of macrophages and immunomodulation. In addition, angiogenic cytokines secreted by ADSCs can enhance the establishment of micro‐capillary networks, which ensure the supply of nutrients and oxygen. These activities may synergistically promote fibrotic lung tissue remodeling.

It is well established that ADSCs exert their therapeutic effects through paracrine activity. The angiogenic cytokines secreted by ADSCs, such as VEGF and PDGF, facilitate endothelial cell proliferation and promote vascularization. Early establishment of micro‐capillary networks provides an adequate supply of nutrients and oxygen, which is conducive to endogenous repair.^[^ ^213^ ^]^ In addition, ADSCs release anti‐inflammatory cytokines including IL‐4 and IL‐10, which induce a phenotypic switch in macrophages from the inflammatory M1 state to the anti‐inflammatory M2 state, eventually leading to immunomodulation.^[^ ^214^ ^]^ These activities enhance fibrotic lung tissue remodeling by ameliorating the lung microenvironment, which may explain the improved prognosis of COVID‐19 patients. Among the numerous therapeutic approaches used to reduce the massive inflammatory phase of SARS‐CoV‐2 infection, ADSCs may serve as a potential weapon against COVID‐19 (**Figure** **12**).

Recently, Sánchez‐Guijo et al. conducted a clinical trial to investigate the safety and efficacy of ADSCs in COVID‐19 patients.^[^ ^215^ ^]^ They treated 13 adult COVID‐19 patients who were under invasive mechanical ventilation with allogeneic ADSCs obtained from healthy donors. Clinical improvement, with a decrease in inflammatory parameters and an increase in lymphocytes, was observed in nine patients during a median follow‐up of 16 d, without significant adverse events. Although the favorable response did not exclusively result from the effect of ADSCs considering other concomitant treatments, this study suggested that the administration of ADSCs is potentially useful in COVID‐19 patients. Moreover, the safety and efficacy of nebulized exosomes derived from allogeneic ADSCs for treating COVID‐19 have been demonstrated.^[^ ^216^ ^]^ It is necessary to further investigate ADSC‐based therapies before specific therapeutics for COVID‐19 become available.

## 5 Conclusion and Future Perspectives

ADSCs represent a promising therapeutic tool in regenerative medicine. Although the mechanisms underlying their regenerative effects have not yet been fully elucidated, numerous preclinical studies (Table S1, Supporting Information) and clinical trials (**Table** **1**) have revealed the therapeutic potential of ADSCs in fat grafting, wound healing, bone regeneration, skeletal muscle repair, tendon reconstruction, cartilage regeneration, cardiac repair, and nerve regeneration. The potential use of ADSCs in the treatment of COVID‐19 is also discussed. This review provides an update on the landscape of ADSCs applications in regenerative medicine and highlights the broad prospects of this field.

**Table 1:** Recent clinical trials of ADSCs for regenerative therapies

Application	Participants	Methods	Follow‐up time	Results	Refs.
Fat grafting	10 healthy participants	The autologous ADSC‐enriched fat grafts and fat graft without ADSCs enrichment were subcutaneously transplanted into the upper arm	121 d	The ADSC‐enriched fat grafts had significantly higher fat survival than control grafts, without serious adverse events	[94]
Wound healing	59 patients with diabetic foot ulcers	30 patients were treated with allogeneic ADSCs–hydrogel complex, while 29 patients received polyurethane film treatment	12 weeks	Allogeneic ADSCs–hydrogel complex significantly promoted wound closure of diabetic foot ulcers	[107]
	296 patients with skin wounds caused by burn or crush injury.	146 patients received allogeneic ADSCs treatment, while 150 patients received conventional dressing with normal saline	10 d	ADSCs increased the granulation tissue coverage rate and promoted wound healing, without adverse events	[108]
Bone regeneration	13 patients with craniomaxillofacial hard‐tissue defects	Autologous ADSCs were seeded onto either bioactive glass or *β*‐tricalcium phosphate scaffolds and transplanted into bone defect area	12–52 months	Successful bone regeneration was observed in 10 of the 13 cases	[135]
	Five cranial defect patients	Patients received cranioplasties using autologous ADSCs, beta‐tricalcium phosphate granules and supporting meshes	6 years	The long‐term clinical results were not satisfactory, partially due to resorption of the graft, tumor recurrence or late infection	[136]
Skeletal muscle repair	18 patients with sphincter defects	9 patients underwent allogeneic ADSCs injection during repair surgery treatment, while 9 patients underwent repair surgery alone	2 months	The ADSCs injection during repair surgery caused the replacement of fibrous tissue and improved the contractile function	[147]
Tendon reconstruction	70 patients with full‐thickness rotator cuff tear	35 patients underwent arthroscopic rotator cuff repair with autologous ADSCs injections, while 35 patients underwent repair surgery alone	At least 12 months	The ADSCs injection during rotator cuff repair significantly decreased the retear rate, whereas the function of repaired tissue was similarly ameliorated in both groups	[160]
	44 patients with degenerative posterosuperior rotator cuff tear	22 patients underwent arthroscopic rotator cuff repair augmentation with autologous microfragmented lipoaspirate tissue, while 22 patients underwent repair surgery alone	24 months	The injection of autologous microfragmented adipose tissue effectively promoted the functional rotator cuff repair	[161]
Cartilage regeneration	12 patients with knee osteoarthritis	Autologous ADSCs were intra‐articularly administered	6 months	The injection of ADSCs provided significant improvement in pain levels and function	[177]
	18 patients with knee osteoarthritis	Three groups underwent autologous ADSCs injections at low dose (2 × 10^6^), medium dose (10 × 10^6^), and high dose (50 × 10^6^), respectively	6 months	Functional improvement and pain relief were observed in patients all three groups, whereas statistical significance was detected only for patients treated with the low dose	[178]
Cardiac repair	10 patients with ischemic heart failure	Allogeneic ADSCs were injected into the infarct border zone	6 months	The ADSCs injection ameliorated cardiac function with safety	[190]
	60 patients with refractory angina	40 patients underwent intramyocardial autologous ADSCs injection, while 20 patients underwent saline injection	3 years	The injection of ADSCs improved cardiac symptoms, whereas exercise capacity remained unchanged	[191]
Nerve regeneration	one patient with spinal cord injury	Autologous ADSCs were intrathecally injected	18 months	The individual recovered to American Spinal Injury Association Impairment Scale grade C	[208]

However, the clinical translation of ADSC‐based therapies still faces many challenges, so this paper discusses novel strategies that provide us with opportunities to overcome the limitations of ADSCs. First, ADSCs secretome, especially ADSCs‐Exos, has been demonstrated to be effective regenerative agents for cell‐free therapy and avoid many shortcomings of administering whole cells such as potential tumorigenicity and storage problems. Second, recent advances in 3D bioprinting promote the development of organoids and organ‐on‐a‐chip systems, which mimic the complex functionalities and architectures of in vivo human organs. Traditional cell culture systems and animal models are insufficient to predict the effectiveness of ADSCs in humans, so the introduction of 3D bioprinting may facilitate the clinical translation of ADSC‐based therapies in the near future. 3D bioprinting using ADSCs also makes it possible to construct organ‐level functional 3D tissues. Third, the absence of standard procedures for applying ADSCs leads to varied cell quality, which makes it difficult to compare results obtained from different studies and increases the uncertainty in their clinical efficacy. Optimized and standardized procedures for applying ADSCs at different checkpoints, including donor site selection, isolation procedure, and storage are essential for the widespread use of ADSCs

The future of ADSCs is only beginning. ADSC‐based therapies are expected to become more tissue‐specific in the future. According to the damaged organs of the patients, clinicians can harvest suitable ADSCs subpopulations through standardized isolation procedures or freeze‐thawing processes, then enhance their therapeutic potential by preconditioning or combining them with biofunctional materials before transplantation. Despite the current challenges, opportunities provided by emerging technologies will drive ADSCs to play a more important role in regenerative medicine.

## Conflict of Interest

The authors declare no conflict of interest.

## Supporting information

Supporting Information
