# Supplementary material for: An Update on Adipose‐Derived Stem Cells for Regenerative Medicine: Where Challenge Meets Opportunity
Source: Adv Sci (Weinh). 2023 May 10;10(20):2207334. doi: 10.1002/advs.202207334 (PMC10369252; doi:10.1002/advs.202207334)
Supplement: Supplementary file 1 — Supporting Information [file ADVS-10-2207334-s001.pdf]

ADVANCED SCIENCE
Open Access

Supporting Information

for *Adv. Sci.*, DOI 10.1002/advs.202207334

An Update on Adipose-Derived Stem Cells for Regenerative Medicine: Where Challenge Meets Opportunity

Yi Qin, Gaoran Ge, Peng Yang, Liangliang Wang, Yusen Qiao, Guoqing Pan, Huilin Yang, Jiaxiang Bai, Wenguo Cui* and Dechun Geng**

Supporting Information

An Update on Adipose-derived Stem Cells for Regenerative Medicine: Where Challenge Meets Opportunity

Yi Qin, Gaoran Ge, Peng Yang, Liangliang Wang, Yusen Qiao, Guoqing Pan, Huilin Yang, Jiaxiang Bai, Wenguo Cui*, Dechun Geng**

Y. Qin, G. Ge, P. Yang, Y. Qiao, Prof. H. Yang, J. Bai, Prof. D. Geng
Department of Orthopaedics, The First Affiliated Hospital of Soochow University; Orthopaedic Institute, Medical College, Soochow University, Suzhou, 215006, Jiangsu, China.
E-mail: jxbai1995@163.com (J. Bai); szgengdc@suda.edu.cn (D. Geng)

L. Wang
Department of Orthopaedics, The Affiliated Changzhou No. 2 People's Hospital of Nanjing Medical University, Changzhou, 213000, Jiangsu, China.

Prof. G. Pan
Institute for Advanced Materials, School of Materials Science and Engineering, Jiangsu University, Zhenjiang, 212013, Jiangsu, China.

Prof. W. Cui
Department of Orthopaedics, Shanghai Key Laboratory for Prevention and Treatment of Bone and Joint Diseases, Shanghai Institute of Traumatology and Orthopaedics, Ruijin Hospital, Shanghai Jiao Tong University School of Medicine, Shanghai, 200025, China.
E-mail: wgcui@sjtu.edu.cn

Y. Qin, **G. Ge** and **P. Yang** contributed equally to this work

Table S1. Recent Preclinical Studies of ADSCs for Regenerative Therapies.

Application	Models	Methods	Results	Reference
**Fat grafting**	A fat graft TLR4(-/-) and Nrf2(-/-) mice model	ADSCs were implanted with adipose tissue	ADSCs increased the survival rate of fat grafts via crosstalk between the Nrf2 and TLR4 pathways	[1]
	A fat graft nude mice model	ADSCs were transfected with modRNA encoding VEGF and co-transplanted with human fat	$ADSC^{modVEGF}$ enhanced angiogenesis and long-term graft survival	[2]
	A fat graft nude mice model	CD34+CD146+ ADSCs subpopulation with enhanced angiogenic potential were isolated and co-transplanted with adipose tissue	Fat enriched CD34+CD146+ ADSCs exhibited improved survival rate with increased expression of proangiogenic factors	[3]
**Wound healing**	A mouse full-thickness skin defect model	Mice were divided into three groups and received ADSCs by topical application, intravenous injection, and intramuscular injection respectively	ADSCs accelerated wound healing independent of their techniques for administration	[4]
	A rat full-thickness skin defect model	ADSCs administered systemically into the vein or locally around the wound	ADSCs administered by intravenous injection promoted wound healing without homing to the wound bed	[5]

	A rat diabetic wound model	ADSCs were incorporated into PRP and injected subcutaneously	ADSCs combined with PRP induced a higher wound closure rate with enhanced neovascularization	[6]
	A rat diabetic wound model	ADSCs were preconditioned with PBM and grafted into the wound	ADSCs preconditioned with PBM significantly promoted the wound healing both in vitro and in vivo	[7]
	An ovine burn wound model	ADSCs were administered into grafted burn wound locally	ADSCs ameliorated grafted burn wound healing and accelerated wound bed blood flow with higher VEGF expression	[8]
	A mouse healing-impaired wound model	ADSCs sheets were fabricated through stimulation with L-ascorbate 2-phosphate	ADSC sheets promoted wound healing with reduced scar formation	[9]
**Bone regeneration**	A mouse femoral fracture model	ADSCs were transduced to express FGF and injected to fracture sites	ADSCs overexpressing FGF accelerated fracture healing by facilitating the remodeling of collagen into mineralized callus	[10]
	A mouse tibial infection model	ADSCs were administered into bone defect area after sufficient debridement of infected bones	ADSCs restored bone regeneration after osteomyelitis via upregulation of osteoblastogenesis, and downregulation of B cells and osteoclasts	[11]
	A rat radial defect model	ADSCs were transplanted with HDB	ADSCs-HDB composites showed a strong osteogenic ability	[12]

	A rat femoral defect model	Osteogenically induced ADSCs were combined with MBG scaffolds prevascularized by seeding with endothelial-induced ADSCs	The strategy of time-phase sequential utilization of ADSCs on MBG scaffolds promoted better bone formation	[13]
	A mouse calvarial defect model	ADSCs were assembled with PDGF and biomineral coated fibers to form spheroids and implanted into defect area	ADSCs spheroids incorporating PDGF and biominerals exhibited greater endothelial lineage mRNA expression and osteogenic capability	[14]
	A mouse calvarial defect model	ADSCs were assembled with adenosine and polydopamine coated fibers to form spheroids and implanted to defect area	ADSCs spheroids impregnated with engineered fibers can enable adenosine delivery and promote bone regeneration with enhanced osteogenic differentiation.	[15]
**Skeletal muscle repair**	A rat muscle injury model	ADSCs were pretreated with IL-4 and SDF-1 and transplanted into injured muscles	Cytokine-pretreated ADSCs showed an increased ability to improve skeletal muscle regeneration	[16]
	A mouse muscle ischemia model	ADSCs and HIF-1α-silenced ADSCs were intramuscularly injected into the ischemic muscle	ADSCs promoted ischemic muscle regeneration by inducing M2 macrophage polarization via the HIF-1α/IL-10 pathway, while the therapeutic effect decreased in HIF-1α-silenced ADSCs	[17]

	A mouse muscle injury model	ADSC after implantation were tracked by optical projection tomography	ADSCs ameliorated skeletal muscle regeneration without direct participation in muscle fiber formation	[18]
	A rat muscle ischemia-reperfusion injury model	Co-application of ADSCs and ECSW was conducted	ECSW-ADSCs treatment appeared more effective than either treatment alone in skeletal muscle repair	[19]
	A rabbit anal sphincter injury model	Co-application of ADSCs and a low-level laser therapy was conducted	Laser-ADSCs treatment was superior to either one alone in repair of anal sphincter injury	[20]
**Tendon reconstruction**	A rat achilles excision defect model	Tenogenically differentiated ADSCs, undifferentiated ADSCs, or hydrogel alone were injected into the achilles excision defect respectively	ADSCs, especially those tenogenically differentiated improved the biomechanical properties of repaired tendon more than hydrogel alone	[21]
	A rat rotator cuff tear model	ADSCs sheets were transplanted to the rotator cuff tear area	ADSCs sheets significantly enhanced the biomechanical properties of repaired rotator cuff	[22]
	A rabbit patellar tendon defect model	ADSCs sheets stimulated by GDF-5 were combined with nanoyarn scaffolds and implanted into patellar	The GDF-5-induced ADSCs sheets expressed higher tenogenesis-related markers and promoted functional tendon	[23]

		tendon defect area	regeneration	
**Cartilage regeneration**	A rat osteochondral defect model	ADSCs were transplanted with an injectable GCS/DF-PEG hydrogel	The ADSCs and GCS/DF-PEG hydrogel complexes showed obvious cartilage regeneration	[24]
	A rat osteoarthritis model	ADSCs were transplanted with an injectable AM hydrogel	ADSCs and AM hydrogel exhibited synergistic anti-inflammatory and chondroprotective effects	[25]
	A rabbit osteochondral defect model	ADSCs and IGF-1 were transplanted with coacervate-embedded composite hydrogels	The dual delivery platform was able to induce chondrogenic differentiation of embedded ADSCs and promote cartilage regeneration effectively.	[26]
	A rabbit osteochondral defect model	CD146+ ADSCs were combined with ACECM and implanted into defect area	The CD146+ ADSCs-ACECM composites exhibited an excellent inflammation-modulating property and promoted better cartilage regeneration	[27]
	A pig osteochondral defect model	ADSCs were combined with ACECM and transplanted into defect area	The ADSCs-ACECM composites successfully repaired the cartilage defect	[28]
**Cardiac**	A mouse MI model	ADSCs reprogrammed with six transcription factors	The reprogrammed ADSCs exhibited higher survival rate and	[29]

**repair**		(Baf60c, Gata4, Gata6, Klf15, Mef2a, and Myocd) were implanted into the infarct border zone	significantly reduced the infarction scar area	
	A rat MI model	ADSCs were transplanted with an injectable conductive hydrogen sulfide-releasing hydrogel	The ADSCs-loaded conductive hydrogen sulfide-releasing hydrogel system ameliorated the harsh microenvironment and improved the cardiac functions remarkablely	[30]
	A rat MI model	ADSCs combined with NRG-MPs were injected into the infarct border zone	ADSC-NRG-MPs improved cell engraftment and neoangiogenesis, favoring a synergy for inducing overall cardiac remodeling	[31]
	A rat MI model	ADSCs and plasmid DNA-eNOs were transplanted with an injectable conductive hydrogel	The ADSCs-plasmid DNA-hydrogel system increased the expression of eNOs in MI tissue and significantly ameliorated the cardiac functions	[32]
	A mouse MI model	ADSCs were injected into the infarct border zone with CTRP9	CTRP9 promoted ADSCs survival, stimulated ADSCs migration, and attenuated cardiomyocyte cell death	[33]
**Nerve**	A rat sciatic nerve injury	ADSCs were induced by FGF9 and administered	The FGF9-induced ADSCs participated in myelin sheath	[34]

**regeneration**	model	directly	formation and facilitated axonal regrowth to promote nerve regeneration	
	A rat sciatic nerve defect model	ADSCs sheets with activation of neurotrophic genes were administered directly	The functionalized ADSCs sheets stimulated axon regeneration, remyelination, and nerve reinnervation	[35]
	A rat sciatic nerve defect model	ADSCs were loaded with pl-CSMCs and injected into NGCs	The microcarrier-based ADSCs transplantation improved the repair effect of NGCs effectively	[36]
	A rat sciatic nerve injury model	ADSCs were loaded with SPIONs and magnetically recruited to the injured site	Magnetic targeted ADSCs therapy promoted cell recruitment at the injured site and ameliorated recovery over ADSCs treatment alone	[37]
	A rat spinal cord injury model	ADSCs were administrated with combination of low-level laser	Combination of ADSCs and laser improved motor function recovery, hyperalgesia, and allodynia more than ADSCs alone, with increased number of axons around cavity	[38]
	A rat spinal cord injury model	ADSCs were transplanted with a CaNeu hydrogel	The CaNeu-hydrogel-mediated ADSCs delivery promoted axonal growth and functional repair	[39]

Reference

[1] X. Chen, L. Yan, Z. Guo, Z. Chen, Y. Chen, M. Li, C. Huang, X. Zhang, L. Chen, *Cell Death Dis* **2016**, 7, e2369.

[2] F. Yu, N. Witman, D. Yan, S. Zhang, M. Zhou, Y. Yan, Q. Yao, F. Ding, B. Yan, H. Wang, W. Fu, Y. Lu, Y. Fu, *Stem Cell Res Ther* **2020**, 11, 490.

[3] M. R. Borrelli, R. A. Patel, C. Blackshear, S. Vistnes, N. M. Diaz Deleon, S. Adem, A. H. Shen, J. Sokol, A. Momeni, D. Nguyen, M. T. Longaker, D. C. Wan, *Stem Cells Translational Medicine* **2020**, 9, 1389.

[4] H. Kim, M. R. Hyun, S. W. Kim, *Stem Cells Int* **2019**, 2019, 2745640.

[5] K. Kallmeyer, D. André-Lévigne, M. Baquié, K.-H. Krause, M. S. Pepper, B. Pittet-Cuénod, A. Modarressi, *Stem Cells Translational Medicine* **2020**, 9, 131.

[6] X. Ni, X. Shan, L. Xu, W. Yu, M. Zhang, C. Lei, N. Xu, J. Lin, B. Wang, *Stem Cell Research & Therapy* **2021**, 12, 226.

[7] H. Ahmadi, A. Amini, F. Fadaei Fathabady, A. Mostafavinia, F. Zare, R. Ebrahimpour-Malekshah, M. N. Ghalibaf, M. Abrisham, F. Rezaei, R. Albright, S. K. Ghoreishi, S. Chien, M. Bayat, *Stem Cell Research & Therapy* **2020**, 11, 494.

[8] O. Fujiwara, A. Prasai, D. Perez-Bello, A. El Ayadi, I. Y. Petrov, R. O. Esenaliev, Y. Petrov, D. N. Herndon, C. C. Finnerty, D. S. Prough, P. Enkhbaatar, *Burns Trauma* **2020**, 8, tkaa009.

[9] J. Yu, M. Y. Wang, H. C. Tai, N. C. Cheng, *Acta Biomater* **2018**, 77, 191.

[10] H. Zhang, A. Kot, Y.-A. E. Lay, F. A. Fierro, H. Chen, N. E. Lane, W. Yao, *Stem Cells Translational Medicine* **2017**, 6, 1880.

[11] J. M. Wagner, F. Reinkemeier, C. Wallner, M. Dadras, J. Huber, S. V. Schmidt, M. Drysch, S. Dittfeld, H. Jaurich, M. Becerikli, K. Becker, N. Rauch, V. Duhan, M. Lehnhardt, B. Behr, *Stem Cells Translational Medicine* **2019**, 8, 1084.

[12] J. Liu, P. Zhou, Y. Long, C. Huang, D. Chen, *Stem Cell Research & Therapy* **2018**, 9, 79.

[13] J. Du, P. Xie, S. Lin, Y. Wu, D. Zeng, Y. Li, X. Jiang, *ACS Appl Mater Interfaces* **2018**, 10, 28340.

[14] J. Lee, S. Lee, T. Ahmad, S. K. Madhurakkat Perikamana, J. Lee, E. M. Kim, H. Shin, *Biomaterials* **2020**, 255, 120192.

[15] T. Ahmad, H. Byun, J. Lee, S. K. Madhurakat Perikamana, Y. M. Shin, E. M. Kim, H. Shin, *Biomaterials* **2020**, 230, 119652.

[16] M. Zimowska, K. Archacka, E. Brzoska, J. Bem, A. M. Czerwinska, I. Grabowska, P. Kasprzycka, E. Michalczewska, I. Stepaniec, M. Soszynska, K. Ilach, W. Streminska, M. A. Ciemerych, *International Journal of Molecular Sciences* **2020**, 21.

[17] J. Liu, P. Qiu, J. Qin, X. Wu, X. Wang, X. Yang, B. Li, W. Zhang, K. Ye, Z. Peng, X. Lu, *Stem Cells (Dayton, Ohio)* **2020**, 38, 1307.

[18] A. Gorecka, S. Salemi, D. Haralampieva, F. Moalli, D. Stroka, D. Candinas, D. Eberli, L. Brügger, *Stem Cell Research & Therapy* **2018**, 9, 195.

[19] T.-C. Yin, R.-W. Wu, J.-J. Sheu, P.-H. Sung, K.-H. Chen, J. Y. Chiang, S.-K. Hsueh, W.-J. Chung, P.-Y. Lin, S.-L. Hsu, C.-C. Chen, C.-Y. Chen, P.-L. Shao, H.-K. Yip, *Oxid Med Cell Longev* **2018**, 2018, 6012636.

[20] A. Sarveazad, A. Babahajian, A. Yari, C. K. Rayner, M. Mokhtare, A. Babaei-Ghazani, S. Agah, B. Mahjoubi, J. Shamseddin, M. Yousefifard, *Stem Cell Research & Therapy* **2019**, 10, 367.

[21] J. B. Norelli, D. P. Plaza, D. N. Stal, A. M. Varghese, H. Liang, D. A. Grande, *J Tissue Eng* **2018**, 9, 2041731418811183.

[22] M. J. Shin, I. K. Shim, D. M. Kim, J. H. Choi, Y. N. Lee, I.-H. Jeon, H. Kim, D. Park, E. Kholinne, H.-S.

Yang, K. H. Koh, *Am J Sports Med* **2020**, 48, 3347.

[23] S. Chen, J. Wang, Y. Chen, X. Mo, C. Fan, *Mater Sci Eng C Mater Biol Appl* **2021**, 119, 111506.

[24] J. Yang, X. Jing, Z. Wang, X. Liu, X. Zhu, T. Lei, X. Li, W. Guo, H. Rao, M. Chen, K. Luan, X. Sui, Y. Wei, S. Liu, Q. Guo, *Front Bioeng Biotechnol* **2021**, 9, 607709.

[25] M. Bhattacharjee, J. L. Escobar Ivirico, H.-M. Kan, S. Shah, T. Otsuka, R. Bordett, M. Barajaa, N. Nagiah, R. Pandey, L. S. Nair, C. T. Laurencin, *Proc Natl Acad Sci U S A* **2022**, 119.

[26] H. Cho, J. Kim, S. Kim, Y. C. Jung, Y. Wang, B.-J. Kang, K. Kim, *J Control Release* **2020**, 327, 284.

[27] X. Li, W. Guo, K. Zha, X. Jing, M. Wang, Y. Zhang, C. Hao, S. Gao, M. Chen, Z. Yuan, Z. Wang, X. Zhang, S. Shen, H. Li, B. Zhang, H. Xian, Y. Zhang, X. Sui, L. Qin, J. Peng, S. Liu, S. Lu, Q. Guo, *Theranostics* **2019**, 9, 5105.

[28] L. Lu, X. Shang, B. Liu, W. Chen, Y. Zhang, S. Liu, X. Sui, A. Wang, Q. Guo, *Journal of Cellular Physiology* **2021**, 236, 4244.

[29] S. Narita, K. Unno, K. Kato, Y. Okuno, Y. Sato, Y. Tsumura, Y. Fujikawa, Y. Shimizu, R. Hayashida, K. Kondo, R. Shibata, T. Murohara, *iScience* **2022**, 25, 104651.

[30] W. Liang, J. Chen, L. Li, M. Li, X. Wei, B. Tan, Y. Shang, G. Fan, W. Wang, W. Liu, *ACS Appl Mater Interfaces* **2019**, 11, 14619.

[31] P. Díaz-Herráez, L. Saludas, S. Pascual-Gil, T. Simón-Yarza, G. Abizanda, F. Prósper, E. Garbayo, M. J. Blanco-Prieto, *J Control Release* **2017**, 249, 23.

[32] W. Wang, B. Tan, J. Chen, R. Bao, X. Zhang, S. Liang, Y. Shang, W. Liang, Y. Cui, G. Fan, H. Jia, W. Liu, *Biomaterials* **2018**, 160, 69.

[33] W. Yan, Y. Guo, L. Tao, W. B. Lau, L. Gan, Z. Yan, R. Guo, E. Gao, G. W. Wong, W. L. Koch, Y. Wang, X.-L. Ma, *Circulation* **2017**, 136, 2162.

[34] C.-W. Huang, S.-Y. Lu, T.-C. Huang, B.-M. Huang, H. S. Sun, S.-H. Yang, J.-I. Chuang, Y.-Y. Hsueh, Y.-T. Wu, C.-C. Wu, *Theranostics* **2020**, 10, 2817.

[35] M.-N. Hsu, H.-T. Liao, V. A. Truong, K.-L. Huang, F.-J. Yu, H.-H. Chen, T. K. N. Nguyen, P. Makarevich, Y. Parfyonova, Y.-C. Hu, *Theranostics* **2019**, 9, 6099.

[36] Y. Sun, X. Chi, H. Meng, M. Ma, J. Wang, Z. Feng, Q. Quan, G. Liu, Y. Wang, Y. Xie, Y. Zheng, J. Peng, *Bioactive Materials* **2021**, 6, 3987.

[37] P. A. Soto, M. Vence, G. M. Piñero, D. F. Coral, V. Usach, D. Muraca, A. Cueto, A. Roig, M. B. F. van Raap, C. P. Setton-Avruj, *Acta Biomaterialia* **2021**, 130, 234.

[38] A. Sarveazad, A. Janzadeh, G. Taheripak, S. Dameni, M. Yousefifard, F. Nasirinezhad, *Stem Cell Research & Therapy* **2019**, 10, 183.

[39] X. Yuan, W. Yuan, L. Ding, M. Shi, L. Luo, Y. Wan, J. Oh, Y. Zhou, L. Bian, D. Y. B. Deng, *Biomaterials* **2021**, 279, 121190.
